# Exploring perceptions and operational considerations for use of a smartphone application to self-monitor blood pressure in pregnancy in Lombok, Indonesia: protocol for a qualitative study

**DOI:** 10.1136/bmjopen-2023-073875

**Published:** 2023-12-18

**Authors:** Tigest Tamrat, Yuni Dwi Setiyawati, Maria Barreix, Mergy Gayatri, Shannia Oktaviana Rinjani, Melissa Paulina Pasaribu, Antoine Geissbuhler, Anuraj H Shankar, Özge Tunçalp

**Affiliations:** 1 UNDP/UNFPA/UNICEF/WHO/World Bank Special Programme of Research Development and Research Training in Human Reproduction (HRP), Department of Sexual and Reproductive Health and Research, World Health Organization, Geneva, Switzerland; 2 University of Geneva, Geneva, Switzerland; 3 Summit Institute for Development, Mataram, Indonesia; 4 Brawijaya University, Malang, Indonesia; 5 HI5lab, Faculty of Medicine, University of Geneva, Geneva, Switzerland; 6 Oxford University Clinical Research Unit-Indonesia, Jakarta, Indonesia

**Keywords:** blood pressure, information technology, maternal medicine, prenatal diagnosis, qualitative research, self care

## Abstract

**Introduction:**

Hypertensive disorders of pregnancy (HDP) are a leading cause of maternal deaths globally and require close monitoring of blood pressure (BP) to mitigate potential adverse effects. Despite the recognised need for research on self-monitoring of blood pressure (SMBP) among pregnant populations, there are very few studies focused on low and middle income contexts, which carry the greatest burden of HDPs. The study aims to understand the perceptions, barriers, and operational considerations for using a smartphone software application to perform SMBP by pregnant women in Lombok, Indonesia.

**Methods and analysis:**

This study includes a combination of focus group discussions, in-depth interviews and workshop observations. Pregnant women will also be provided with a research version of the smartphone BP application to use in their home and subsequently provide feedback on their experiences. The study will include pregnant women with current or past HDP, their partners and the healthcare workers involved in the provision of antenatal care services within the catchment area of six primary healthcare centres. Data obtained from the interviews and observations will undergo thematic analyses using a combination of both inductive and deductive approaches.

**Ethics and dissemination:**

The study was approved by the World Health Organization (WHO) and Human Reproduction Programme (HRP) Research Project Review Panel and WHO Ethical Review Committee (A65932) as well as the Health Research Ethics Committee, Faculty of Medicine, Universitas Mataram in Indonesia (004/UN18/F7/ETIK/2023).

Findings will be disseminated through research publications and communicated to the Lombok district health offices. The analyses from this study will also inform the design of a subsequent impact evaluation.

**STRENGTHS AND LIMITATIONS OF THIS STUDY**

- This study addresses a research gap identified by the WHO and expands on the geographical scope of available evidence on self-monitoring of blood pressure (SMBP) beyond high-income settings.
- The study leverages a novel approach of a smartphone-based blood pressure (BP) measurement to examine the intersection of digital health, maternal health, self-care and health systems.
- To provide a holistic perspective, the study will obtain insights from a diverse set of participants, including pregnant women, their partners and the health workforce across different levels of the health system (e.g. community health workers, midwives, doctors, managers).
- A limitation of this study is that pregnant women will not be actively self-monitoring their BP to detect clinical changes but rather undergo the process to understand their perceptions of SMBP and implementation considerations.
- This study will inform subsequent impact evaluations and complements existing initiatives within the research site on the use of digital tools towards identification and management of hypertensive disorders of pregnancy.

## Introduction

Hypertensive disorders of pregnancy (HDPs) are one of the most common pregnancy complications and a leading cause of maternal mortality and morbidity.^1–5^ Approximately 14% of all maternal deaths globally are attributed to HDPs, with the majority in low and middle-income contexts.^2 3^ HDPs include chronic hypertension, gestational hypertension, pre-eclampsia and chronic hypertension with superimposed pre-eclampsia.^1 5^ Chronic hypertension consists of elevated blood pressure (BP) that is identified prior to the pregnancy or detected within the first 20 weeks.^1^ Gestational hypertension is considered to be induced during a pregnancy with the onset of elevated BP after 20 weeks.^1^ Pre-eclampsia is characterised by elevated BP in the presence of either proteinuria or other new onset maternal organ dysfunction, neurological conditions or fetal growth restriction.^6^ It is a precursor to eclampsia, a life-threatening condition which accounts for the majority of deaths attributed to HDPs.^2 4^

The World Health Organization (WHO) recommends routine BP measurement during pregnancy and at every point of contact in the provision of antenatal care (ANC) as part of efforts to improve maternal health.^7 8^ In certain contexts, pregnant women identified with HDP may require additional follow-up and monitoring in order to mitigate potential adverse outcomes.^9^ Furthermore, as the underlying cause of pre-eclampsia is unknown and onset may be triggered in between clinical consultations, close monitoring of BP beyond the health facility is critical.^5 10 11^

### Self-monitoring of blood pressure

Self-monitoring of blood pressure (SMBP), also known as home-based BP monitoring, is well researched in the general population^12–17^ with a WHO recommendation in 2013 supporting its appropriate and affordable use.^18^ Cited advantages include improved BP control and reduced anxiety associated with ‘white coat’ hypertension.^10–12 15 17^ However, studies also highlight challenges associated with the variety of devices used within home settings, reliability of readings and calibration requirements, as well as ensuring guidance to individuals on interpreting results, dealing with variations of readings and appropriate actions to be taken.^19 20^

SMBP during pregnancy is an emerging approach recommended by WHO in 2021 ‘as an additional option to clinic blood pressure monitoring by healthcare workers during antenatal contacts only, for individuals with hypertensive disorders of pregnancy’.^21^ Although there is a substantial literature base on SMBP in non-pregnant populations,^12–16 19 20 22–29^ the evidence specific to pregnancy is still nascent^11 30–33^ and limited for low- and middle-income countries (LMIC) where there remains a high burden of HDPs.^34^ To date, studies that explore SMBP among pregnant women span Australia, Canada, Japan, UK, New Zealand and USA,^35 36^ with one study in South Africa focused on knowledge and attitudes of SMBP among patients with pre-eclampsia.^34^ A Cochrane systematic review on the different approaches to SMBP in pregnancy indicated uncertainties in the benefits of SMBP of hypertensive pregnancies due to limited number of studies.^37^ Other systematic reviews on SMBP during antenatal and postpartum periods demonstrated its feasibility but no difference in clinical outcomes,^38^ while another meta-analyses reported reductions in ANC visits, hospital admissions and diagnosis of pre-eclampsia.^39^ All systematic reviews highlighted the lack of studies from LMICs and the need for more evidence, particularly across lower resource settings.^37–39^

Among the available studies, the Blood Pressure Self-Monitoring in Pregnancy (BuMP) research initiative conducted in the UK, offers one of the most comprehensive methods for evaluating feasibility and effectiveness.^30 31 40 41^ For the feasibility assessment, women between 12 and 16 weeks gestational age and identified to be at risk for pre-eclampsia were recruited to self-monitor their BP using an automated BP monitor (Microlife WatchBP Home validated for use in pregnancy and pre-eclampsia). Study participants had the option to also text their readings and receive automated responses and text prompts.^31^ Women reported that the approach was reassuring and made them more informed about HDPs; however, study authors noted the limited generalisability of the findings across different socioeconomic backgrounds.^31^ This assessment was followed by a prospective cohort study of approximately 200 women and demonstrated that women at risk for pre-eclampsia were able to detect gestational hypertension in-between clinical visits.^30^ The study also explored healthcare workers’ perspectives and noted their concerns in reconciling the autonomy and reassurance that women may gain in exchange with the caution and uncertainty that self-monitoring requires.^41^ More recently, the BuMP study conducted a randomised clinical trial (RCT) among 2441 pregnant individuals at increased risk for pre-eclampsia, in which there was no statistically significant differences in early detection of pre-eclampsia between SMBP and standard care groups.^42^ Similarly, there was also no statistically significant differences among pregnant individuals with chronic or gestational hypertension.^43^

Another RCT, also conducted in the UK, enrolled pregnant women with chronic hypertension or gestational hypertension for SMBP using a calibrated BP measurement device (Microlife WatchBP Home); women with pre-eclampsia were excluded from this study.^44^ Study participants reported their second of two BP readings via text, an application designed for the study or noted it on paper to be reviewed at their ANC contact.^44^ In response to their text message or the mobile application, participants received immediate automated responses. Although there was no difference in the mean systolic BP measurements or medication adherence, participants demonstrated willingness and acceptability for self-monitoring.^44^

### Potential for digital technology to facilitate SMBP

Advances in digital technology in LMICs, primarily through penetration of mobile devices,^45^ provide an opportunity to expand SMBP and other health monitoring services. While the catalyst for SBPM in high-income settings was spurred by the rise of automated cuffs,^12^ this has not gained traction in settings in which there is already a limited availability of automated equipment for facility based use.^46^ Furthermore, BP measurement devices require additional validation specific to pregnant populations.^37 47^

**Figure 1:**
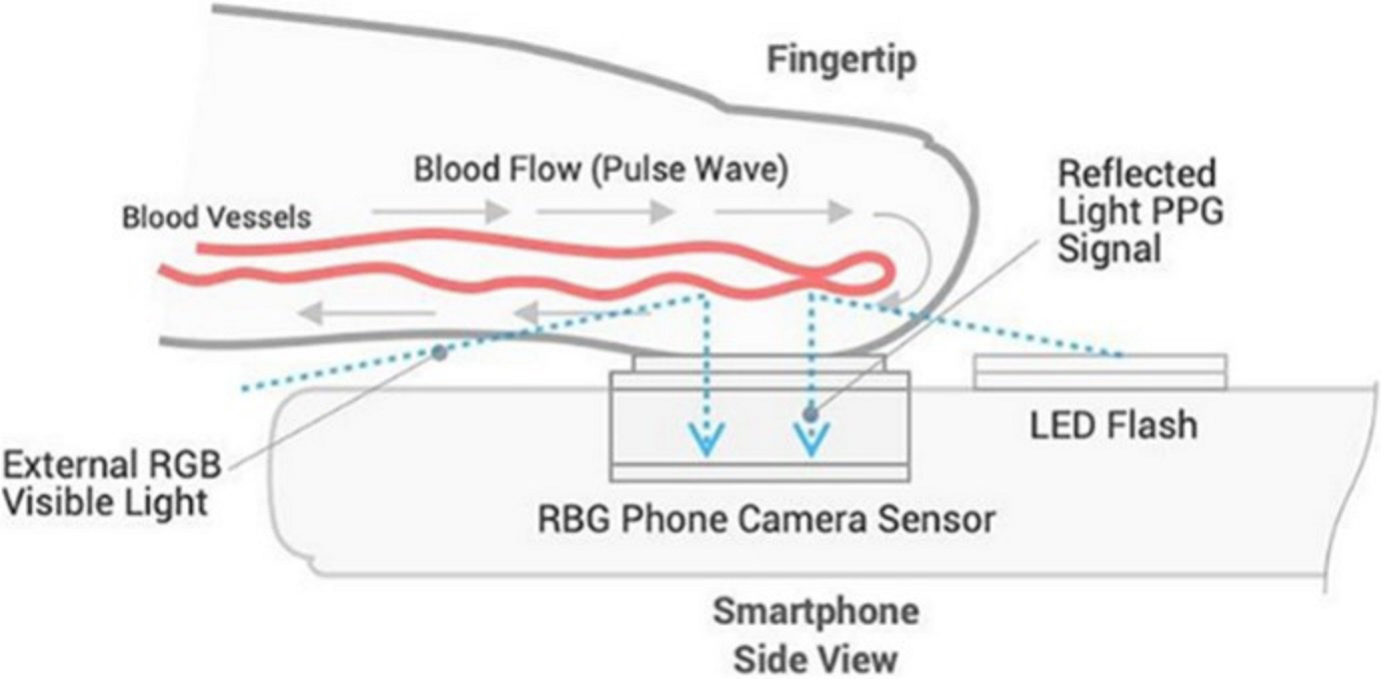
Optical pulse wave technology underlying OptiBP smartphone application.^49^ PPG, photoplethysmographic. LED, Light-Emitting Diode a form of light for flash on camera; RBG, red, blue, green for depicting color images on smartphones.

As an emerging alternative to measuring BP, OptiBP is a software application that uses smartphone cameras to capture optical pulse-waves of the fingertip and estimate BP readings (figure 1). The OptiBP application runs on Android OS V.8.1 and leverages the smartphone camera to record photoplethysmographic optical pulse waves derived from blood volume changes at the fingertips and estimate BP values using an algorithm.^48 49^ This software based algorithm has undergone validation in Switzerland, in which it demonstrated concordance against reference BP measurements^49 50^; a complementary validation study of OptiBP was conducted in accordance with the international regulatory standards in Tanzania, South Africa and Bangladesh. This study also included a targeted assessment on the accuracy of BP measurement among pregnant populations to account for the maternal cardiovascular changes on the detection of optical pulse waves.^51 52^ Findings from this multi-site study demonstrated accuracy against International Organisation for Standardisation (ISO) benchmarks, including among pregnant populations, except in Bangladesh for systolic BP ISO criterion 2.^52 53^ A similar validation study was also conducted in Indonesia among pregnant populations in West Lombok and East Lombok in West Nusa Tenggara Province.^54^

### Aims and objectives

The newfound capability of using a smartphone application for BP measurement presents an opportunity for broadening SMBP to LMIC settings, where the burden of HDPs is the greatest and mobile technologies have gained widespread use for maternal health.^51 55–63^ Using these advances in BP monitoring, this research addresses evidence gaps identified in the WHO guideline on self-care interventions regarding the feasibility, benefits and harms of SMBP among individuals with HDP, particulary for non high income settings.^21^ This study explores the perceptions, barriers and operational considerations for SMBP via a smartphone application (OptiBP) in Lombok, Indonesia. The first objective of the study is to examine pregnant women’s, their partners and healthcare workers’ attitudes towards SMBP in general and specifically regarding the use of a smartphone application for SMBP. In addition, we will analyze the implementation requirements for SMBP via a smartphone through user-centred design approaches to adapt for health literacy and workflow integration requirements.

## Methods and analysis

### Study design and setting

This study will use a combination of qualitative methods, including focus group discussions (FGD), in-depth interview (IDI) and workshop observations to triangulate findings on perceptions and operational considerations for SMBP by pregnant women. The study will include pregnant women, their partners and the healthcare workers involved in the provision and management of ANC services to obtain a holistic understanding of the dynamics across health system actors. To derive concrete operational considerations, selected pregnant women will also be provided with a research version of the OptiBP smartphone application to use in their home setting and subsequently provide feedback on their experiences. The study will be conducted within the catchment area of six primary healthcare facilities in West Lombok district Lombok, Indonesia. Data collection began in mid-February 2023 and expected to be completed by May 2023.

### Participant selection

The main population for this study is pregnant women with current or past HDP. Eligible participants will be drawn from pregnant women coming to receive ANC at the selected study facilities and registered in the ANC digital module, which is a digital health record tracking and decision support system that was implemented independently of this study.^64^ Following approval from the heads of health facilities to review the digital health records for sampling purposes, the study team will select pregnant women for screening and invitation to participate in the study. A subset of the partners of participating pregnant women will be selected following the consent of the pregnant woman to contact her partner.

A diverse range of health workforce cadres, including community health workers, community and facility-based midwives, obstetrician/gynaecologists and health system managers will also be included to compliment the findings from pregnant women. Table 1 provides an overview of the participants and sampling frame.

**Table 1:** Sampling frame

Activity/method	Pregnant women with HDP		Partners of pregnant women	Health workers/manager				
	<30 years	≥30 years		CHWs	Nurse-midwives (community)	Nurse-midwives (facility)	Doctors	Health system managers
FGD on perception of SMBP	2–3 FGDs of 5–7	2–3 FGDs of 5–7	2 FGDs of 5–7	4 FGDs of 5–7	2–3 FGDs of 5–7	2–3 FGDs of 5–7	N/A	N/A
IDI on perception of SMBP	4–5	4–5	N/A	4–5	4–5	4–5	3–5	3–5
Intervention adaptation workshops	5–10	5–10	N/A	N/A	N/A	N/A	N/A	N/A

CHWs, community health workers; FGD, focus group discussion; HDP, hypertensive disorders of pregnancy; IDI, in-depth interview; N/A, not applicable; SMBP, self-monitoring of blood pressure.

### Sampling and recruitment

The study will use purposive sampling of pregnant women with a current diagnosis or history of HDP (as derived from their medical record in the ANC digital module) and across two age groups to account for potentially differing levels of digital literacy. We will identify women with different educational levels, gestational age and parity through the sampling and collect variables on these factors.

Pregnant women will be approached during their routine ANC contacts once they have completed their consultation. A research assistant will brief women on the study and gauge their interest to participate. The contact information of consenting and eligible pregnant women will be taken, and the research assistant will inform them that they will be contacted if they are selected for the study. Eligibility for pregnant women will be based on the following criteria: confirmed pregnancy (either by healthcare worker or self-administered and documented rapid test), current or history of HDP (derived from the digital record system), greater than 18 years of age or emancipated minor, living within catchment area of selected health facilities, able to read one sentence in Bahasa and having provided written informed consent. Pregnant women will be excluded if they have severe visual impairment that prevents use of the smartphone application (eg, blindness) or absence of index or middle finger on either left or right hand. Partners of pregnant women will be identified through the recruited women after first obtaining consent from the pregnant women to contact their partners. Pregnant women will not be excluded based on ownership of a phone, and the study will provide a smartphone to the participating women who are assigned to use the smartphone application at home.

Healthcare managers will be selected based on their role and oversight in the catchment area. They will be invited to participate in the study and asked for their written informed consent during their work hours. The different cadres of healthcare workers will be identified through District Health Office (DHO) and facility networks under its hierarchical systems, which are Public Health Centres (Puskesmas) and Maternity Clinics (Polindes).

Healthcare workers will be randomly selected to participate in the study based on a list provided by the DHO. The study team will first briefly inform the supervisors and invite the selected healthcare workers to attend an information session where the research staff will elaborate further on the study and ask for their consent to take part in the study. The interviews will take place during work hours after permission has been obtained from the DHO.

The study will invite women to either be part of the FGDs or workshops and randomly allocate to the two groups. Participants for the IDIs will be sampled from the FGDs to examine their feedback in greater detail. The sample size for pregnant women and healthcare workers will be based on requirements for reaching saturation, as well as using an emergent sampling design, in which participants will be recruited as the study progresses.^65^

### Methods of approach

#### Interviews

FGDs and IDIs will be used to examine potential barriers and motivators for SMBP, as well as how this may influence changes in pregnant women’s interactions with healthcare workers. As partners and family members of pregnant women can influence access and use of ANC services, their perceptions of SMBP will also be used to obtain a comprehensive understanding of the considerations for SMBP and any related care.^66^

#### Observation workshops

In addition to the FGDs and IDIs, observation workshops will be conducted to review the smartphone application interface and determine adaptation requirements for enhancing its usability in accordance with health literacy levels and ensuring integration with health system and data workflows. The workshop will have two parts. First, we will introduce a research version of the smartphone application to selected women and observe initial reactions and feedback. Women will be provided with a smartphone loaded with the application and instructions to explore using it at home twice a day for 1 week before returning to the subsequent workshop, 1 week later. This second workshop will be used to gather feedback on their experiences of use and refine guidance/training support needed for achieving fidelity and requirements for integrating within health system workflows.

The software application provided to the participants will be linked to a de-identified study account with access to the local research team. It will also include a disclaimer stating the measurements are not to be used for clinical decision-making and that participants should contact the health facility and research team if they face any health issues. The application will not be used by the research team or healthcare workers for any clinical decision-making; all BP measurements for service provision will be done through the standard care at facilities.

Both workshops will include structured and unstructured observations of pregnant women engaging with the smartphone application interface and will be used to further inform the decision support prompts (eg, what to do in cases of high BP reading) that are contextually appropriate and resonate with the levels of health literacy. In the structured observations, pregnant women will be asked to interact with the smartphone application prototype across various tasks and scenarios, such as turning on the smartphone application, placing their finger on the camera of the smartphone for taking their BP measurement, interpreting the result/output from the BP measurement and undertaking follow-up actions, when necessary.

### Data collection

For the interviews, a semi-structured topic guide will be provided to trained interviewers conducting the FGDs and IDIs. The topic guide for pregnant individuals will cover areas of pregnancy experience, mobile phone use in pregnancy, knowledge of and experience with taking BP, self-efficacy in taking BP, trust and support for taking BP. Interview guides for the healthcare worker will include topics surrounding experience in providing ANC, perceptions of self-management of pregnancy generally, perceptions of SMBP by pregnant women and data flow and health system linkages. All interview guides will include probes and questions for initiating the interviews and allow for open-ended responses and capturing any reflections that may emerge. Interviews will be recorded, transcribed and translated into English for analysis.

For the observation workshops, a research assistant will document both unprompted reactions, as well as responses to specific tasks with the smartphone application. As a supplement to the observation notes, the study will also embed a screen recorder while pregnant women use the application during the workshop to capture the process flows and interactions with the application, including length of time and frequency of use of different buttons. The study team will also take videos and photos during the observation workshop to record feedback related to interactions with the application.

### Analysis

Recordings will be double transcribed and translated for quality assurance. Data obtained from the interviews and observations will first undergo an initial rapid analysis to flag key themes and considerations, including findings from the existing literature base. This will be followed by detailed analysis and coding using a thematic analysis employing a combination of both inductive and deductive approaches.^65 67^ Each finding will be compared and cross-checked to determine whether it aligns with existing categories or if a new theme should be developed.^65^ The analysis will be conducted by two individuals to ensure inter-rater reliability and then reviewed collectively with the broader research team. Screen recordings of interactions with the application will also be reviewed to identify patterns of use. ATLAS.ti^68^ will be used to manage the coding and analysis of themes from the transcripts.

### Patient and public involvement

The views of patients or the public will be reflected in the analysis of the manuscript and through the co-design workshops of the study.

### Ethics and dissemination

The study was approved by the WHO/Human Reproduction Programme Research Review Panel and WHO Ethical Review Committee (Reference number A65932) as well as the Health Research Ethics Committee, Faculty of Medicine, Universitas Mataram in Indonesia (004/UN18/F7/ETIK/2023).

All eligible participants will receive an information sheet and are required to provide informed written consent before participation. Confidential data will be stored at country level and de-identified transcriptions will be shared with WHO to support the analyses. Personal identifiers will be removed during the transcription of audio recordings and labelled with a de-identified participant identification number. Identifiable data will be kept at the research site in a secure location for a minimum of 5 years before securely disposing of primary research data, as per the guidance from the local ethical review committee.

Participants using the smartphone application at home will be provided with detailed guidance, as well as airtime/SIM cards to contact the research team as needed. The smartphone application will not be used by the research team or healthcare workers for any clinical decision-making, and all BP measurements for service provision will be done through the standard care at facilities using standard sphygmomanometers.

Photos and video recordings will only be taken during training and specific group events, and only after written informed consent. The informed consent will explicitly mention the use of audio-recording and screen recording during the workshop. Unless otherwise consented, use of these will be obscured by blurring or other effects to render persons non-identifiable, and the purpose for use will be restricted to training and implementing processes to make the work or intervention more effective. The company that developed the smartphone application will not have access to any data or transcripts but will have access to the feedback shared during their participation in the workshop.

Findings will be disseminated through research publications and communicated to the DHO. The analyses from this study will also inform the design of a subsequent impact evaluation. In addition, the feedback from workshops may contribute to potential refinements to the smartphone application.

## Supplementary Material

Reviewer comments

Author's manuscript

## References

[1] Mammaro A, Carrara S, Cavaliere A, et al. Hypertensive disorders of pregnancy. J Prenat Med 2009;3:1–5. 22439030 PMC3279097

[2] Duley L. The global impact of pre-eclampsia and eclampsia. Semin Perinatol 2009;33:130–7. 10.1053/j.semperi.2009.02.010 19464502

[3] Say L, Chou D, Gemmill A, et al. Global causes of maternal death: a WHO systematic analysis. Lancet Glob Health 2014;2:e323–33. 10.1016/S2214-109X(14)70227-X 25103301

[4] Abalos E, Cuesta C, Grosso AL, et al. Global and regional estimates of preeclampsia and eclampsia: a systematic review. Eur J Obstet Gynecol Reprod Biol 2013;170:1–7. 10.1016/j.ejogrb.2013.05.005 23746796

[5] World health Organization. WHO recommendations for prevention and treatment of pre-Eclampsia and Eclampsia. Geneva WHO Guidelines Approved by the Guidelines Review Committee; 2011. 23741776

[6] JA24 Pre-eclampsia. In: ICD-11 for mortality and morbidity statistics. Geneva: World Health Organization, Available: https://icd.who.int/ browse11/l-m/en#/http://id.who.int/icd/entity/229121159 [accessed 02 Jul 2023].

[7] Hlongwane TM, Bozkurt B, Barreix MC, et al. Implementing antenatal care recommendations, South Africa. Bull World Health Organ 2021;99:220–7. 10.2471/BLT.20.278945 33716344 PMC7941100

[8] World Health Organization. WHO recommendations on Antenatal care for a positive pregnancy experience. Geneva WHO; 2016. 28079998

[9] Excellence NIfHaC. Hypertension in pregnancy: diagnosis and management -- NICE guideline [Ng133]. United Kingdom; 2019. Available: https://www.nice.org.uk/guidance/ng133/chapter/Recommendations

[10] Mustafa R, Ahmed S, Gupta A, et al. A comprehensive review of hypertension in pregnancy. J Pregnancy 2012;2012:105918. 10.1155/2012/105918 22685661 PMC3366228

[11] Hodgkinson JA, Tucker KL, Crawford C, et al. Is self monitoring of blood pressure in pregnancy safe and effective BMJ 2014;349:g6616. 10.1136/bmj.g6616 25406132

[12] Parati G, Stergiou GS, Asmar R, et al. European society of hypertension practice guidelines for home blood pressure monitoring. J Hum Hypertens 2010;24:779–85. 10.1038/jhh.2010.54 20520631

[13] Imai Y, Obara T, Asamaya K, et al. The reason why home blood pressure measurements are preferred over clinic or ambulatory blood pressure in Japan. Hypertens Res 2013;36:661–72. 10.1038/hr.2013.38 23595050

[14] Roerecke M, Kaczorowski J, Myers MG. Comparing automated office blood pressure readings with other methods of blood pressure measurement for identifying patients with possible hypertension: a systematic review and meta-analysis. JAMA Intern Med 2019;179:351–62. 10.1001/jamainternmed.2018.6551 30715088 PMC6439707

[15] Bo Y, Kwok K-O, Chu KK-Y, et al. Comparison between automated office blood pressure measurements and manual office blood pressure measurements-implications in individual patients: a systematic review and meta-analysis. Curr Hypertens Rep 2021;23:4. 10.1007/s11906-020-01118-1 33452580 PMC7810619

[16] Niiranen TJ, Jula Am Fau - Kantola IM, Kantola Im Fau - Reunanen A, et al. n.d. Comparison of agreement between clinic and home-measured blood pressure in the Finnish population: the Finn-HOME study [0263-6352 Print]. 10.1097/01.hjh.0000239290.94764.81 16877957

[17] McManus RJ, Little P, Stuart B, et al. Home and online management and evaluation of blood pressure (HOME BP) using a digital intervention in poorly controlled hypertension: randomised controlled trial. BMJ 2021;372:m4858. 10.1136/bmj.m4858 33468518 PMC7814507

[18] World Health Organization. Package of essential Noncommunicable (PEN) disease interventions for primary health care in low-resource settings. Geneva, Switzerland; 2013.

[19] Fletcher BR, Hinton L, Hartmann-Boyce J, et al. Self-monitoring blood pressure in hypertension, patient and provider perspectives: a systematic review and thematic synthesis. Patient Educ Couns 2016;99:210–9. 10.1016/j.pec.2015.08.026 26341941

[20] McManus RJ, Ryan A, Greenfield S, et al. Self measurement of blood pressure: a community survey. J Hum Hypertens 2007;21:741–3. 10.1038/sj.jhh.1002217 17476287

[21] WHO Consolidated guideline on self-care interventions for health V.2 sexual and reproductive health and rights. Geneva World Health Organization; 2022. Available: https://www.who.int/publications/i/item/9789240052192 31334932

[22] Monahan M, Jowett S, Lovibond K, et al. Predicting out-of-office blood pressure in the clinic for the diagnosis of hypertension in primary care: an economic evaluation. Hypertension 2018;71:250–61. 10.1161/HYPERTENSIONAHA.117.10244 29203628

[23] Hodgkinson J, Mant J, Martin U, et al. Relative effectiveness of clinic and home blood pressure monitoring compared with ambulatory blood pressure monitoring in diagnosis of hypertension: systematic review. BMJ 2011;342:d3621. 10.1136/bmj.d3621 21705406 PMC3122300

[24] Beyhaghi H, Viera AJ. Comparative cost-effectiveness of clinic, home, or ambulatory blood pressure measurement for hypertension diagnosis in US adults. Hypertension 2019;73:121–31. 10.1161/HYPERTENSIONAHA.118.11715 30571548

[25] Tucker K, Sheppard JP, Stevens R, et al. [OP.6B.04] individual patient data meta-analysis of self-monitoring of blood pressure (BP-smart). J Hypertens (Los Angel) 2016;34:e69–70. 10.1097/01.hjh.0000491517.19347.e5

[26] Bray EP, Holder R, Mant J, et al. Does self-monitoring reduce blood pressure? Meta-analysis with meta-regression of randomized controlled trials. Ann Med 2010;42:371–86. 10.3109/07853890.2010.489567 20504241

[27] Uhlig K, Patel K, Ip S, et al. Self-measured blood pressure monitoring in the management of hypertension: a systematic review and meta-analysis. Ann Intern Med 2013;159:185–94. 10.7326/0003-4819-159-3-201308060-00008 23922064

[28] Ward AM, Takahashi O, Stevens R, et al. Home measurement of blood pressure and cardiovascular disease: systematic review and meta-analysis of prospective studies. J Hypertens 2012;30:449–56. 10.1097/HJH.0b013e32834e4aed 22241136

[29] Niiranen TJ, Hänninen M-R, Johansson J, et al. Home-measured blood pressure is a stronger Predictor of cardiovascular risk than office blood pressure: the Finn-home study. Hypertension 2010;55:1346–51. 10.1161/HYPERTENSIONAHA.109.149336 20385970

[30] Tucker KL, Taylor KS, Crawford C, et al. Blood pressure self-monitoring in pregnancy: examining feasibility in a prospective cohort study. BMC Pregnancy Childbirth 2017;17:442. 10.1186/s12884-017-1605-0 29284456 PMC5745883

[31] Hinton L, Tucker KL, Greenfield SM, et al. Blood pressure self-monitoring in pregnancy (bump) feasibility study; a qualitative analysis of women’s experiences of self-monitoring. BMC Pregnancy Childbirth 2017;17:427. 10.1186/s12884-017-1592-1 29258469 PMC5735874

[32] Tucker KL, Bowen L, Crawford C, et al. The feasibility and acceptability of self-testing for proteinuria during pregnancy: a mixed methods approach. Pregnancy Hypertens 2018;12:161–8. 10.1016/j.preghy.2017.11.009 29242046

[33] Wilson H, Tucker KL, Chisholm A, et al. Self-monitoring of blood pressure in pregnancy: a mixed methods evaluation of a national roll-out in the context of a pandemic. Pregnancy Hypertens 2022;30:7–12. 10.1016/j.preghy.2022.07.006 35933759 PMC9364829

[34] Munyungula J, Shakwane S. Self-monitoring of blood pressure for preeclampsia patients: knowledge and attitudes. Curationis 2021;44:e1–8.:2195. 10.4102/curationis.v44i1.2195 PMC8517804 34636622

[35] Tucker KL, Bankhead C, Hodgkinson J, et al. How do home and clinic blood pressure readings compare in pregnancy Hypertension 2018;72:686–94. 10.1161/HYPERTENSIONAHA.118.10917 30354754 PMC6080884

[36] Aquino M, Munce S, Griffith J, et al. Exploring the use of telemonitoring for patients at high risk for hypertensive disorders of pregnancy in the antepartum and postpartum periods: scoping review. JMIR Mhealth Uhealth 2020;8:e15095. 10.2196/15095 32301744 PMC7195666

[37] Ashworth DC, Maule SP, Stewart F, et al. Setting and techniques for monitoring blood pressure during pregnancy. Cochrane Database Syst Rev 2020;8:CD012739. 10.1002/14651858.CD012739.pub2 32748394 PMC8409325

[38] Yeh PT, Rhee DK, Kennedy CE, et al. Self-monitoring of blood pressure among women with hypertensive disorders of pregnancy: a systematic review. BMC Pregnancy Childbirth 2022;22:454. 10.1186/s12884-022-04751-7 35641913 PMC9152837

[39] Kalafat E, Benlioglu C, Thilaganathan B, et al. Home blood pressure monitoring in the antenatal and postpartum period: a systematic review meta-analysis. Pregnancy Hypertens 2020;19:44–51. 10.1016/j.preghy.2019.12.001 31901652

[40] Dougall G, Franssen M, Tucker KL, et al. Blood pressure monitoring in high-risk pregnancy to improve the detection and monitoring of hypertension (the BUMP 1 and 2 trials): protocol for two linked randomised controlled trials. BMJ Open 2020;10:e034593. 10.1136/bmjopen-2019-034593 PMC7044851 31980512

[41] Hinton L, Hodgkinson J, Tucker KL, et al. Exploring the potential for introducing home monitoring of blood pressure during pregnancy into maternity care: current views and experiences of staff-a qualitative study. BMJ Open 2020;10:e037874. 10.1136/bmjopen-2020-037874 PMC7709507 33262186

[42] Tucker KL, Mort S, Yu L-M, et al. Effect of self-monitoring of blood pressure on diagnosis of hypertension during higher-risk pregnancy: the BUMP 1 randomized clinical trial. JAMA 2022;327:1656–65. 10.1001/jama.2022.4712 35503346 PMC9066279

[43] Chappell LC, Tucker KL, Galal U, et al. Effect of self-monitoring of blood pressure on blood pressure control in pregnant individuals with chronic or gestational hypertension: the BUMP 2 randomized clinical trial. JAMA 2022;327:1666–78. 10.1001/jama.2022.4726 35503345 PMC9066282

[44] Pealing LM, Tucker KL, Mackillop LH, et al. A randomised controlled trial of blood pressure self-monitoring in the management of hypertensive pregnancy. OPTIMUM-BP: a feasibility trial. Pregnancy Hypertens 2019;18:141–9. 10.1016/j.preghy.2019.09.018 31618706

[45] International Telecommunications Union. ICT facts and figures. Geneva; 2015. Available: https://www.itu.int/en/ITU-D/Statistics/Documents/facts/ICTFactsFigures2015.pdf [Accessed 23 Mar 2018].

[46] Betrán AP, Bergel E, Griffin S, et al. Provision of medical supply kits to improve quality of antenatal care in mozambique: a stepped-wedge cluster randomised trial. Lancet Glob Health 2018;6:e57–65. 10.1016/S2214-109X(17)30421-7 29241615 PMC5732314

[47] O’Brien E, Atkins N, Stergiou G, et al. European society of hypertension international protocol revision 2010 for the validation of blood pressure measuring devices in adults. Blood Press Monit 2010;15:23–38. 10.1097/MBP.0b013e3283360e98 20110786

[48] Ghamri Y, Proença M, Hofmann G, et al. Automated pulse oximeter waveform analysis to track changes in blood pressure during anesthesia induction: a proof-of-concept study. Anesth Analg 2020;130:1222–33. 10.1213/ANE.0000000000004678 32287129

[49] Schoettker P, Degott J, Hofmann G, et al. Blood pressure measurements with the optibp smartphone app validated against reference auscultatory measurements. Sci Rep 2020;10:17827. 10.1038/s41598-020-74955-4 33082436 PMC7576142

[50] Degott J, Ghajarzadeh-Wurzner A, Hofmann G, et al. Smartphone based blood pressure measurement: accuracy of the optibp mobile application according to the AAMI/ESH/ISO universal validation protocol. Blood Press Monit 2021;26:441–8. 10.1097/MBP.0000000000000556 34139747 PMC8568326

[51] Su F, Li Z, Sun X, et al. The pulse wave analysis of normal pregnancy: investigating the gestational effects on photoplethysmographic signals. Biomed Mater Eng 2014;24:209–19. 10.3233/BME-130801 24211900

[52] Association for the Advancement of Medical Instrumentation, Association for the Advancement of Medical Instrumentation, Association for the Advancement of Medical Instrumentation, et al. Non-invasive Sphygmomanometers — part 2: clinical investigation of automated measurement type. Arlington, VA, USA, 2013.

[53] Festo C, Vannevel V, Ali H, et al. Accuracy of a smartphone application for blood pressure estimation in Bangladesh. NPJ Digit Med 2023;6:69. 10.1038/s41746-023-00804-z 37069209 PMC10107587

[54] Setiyawati YD, Jannah M, Gayatri M, et al. The accuracy of cuffless optical blood pressure assessment in pregnancy and its potential to improve clinical outcomes. 2023.

[55] Saronga NJ, Burrows T, Collins CE, et al. mHealth interventions targeting pregnancy intakes in low and lower-middle income countries: systematic review. Matern Child Nutr 2019;15:e12777. 10.1111/mcn.12777 30609297 PMC7199073

[56] Ilozumba O, Abejirinde I-O, Dieleman M, et al. Targeting strategies of mHealth interventions for maternal health in low and middle-income countries: a systematic review protocol. BMJ Open 2018;8:e019345. 10.1136/bmjopen-2017-019345 PMC5855310 29478019

[57] Dol J, Richardson B, Tomblin Murphy G, et al. Impact of mobile health interventions during the perinatal period for mothers in low- and middle-income countries: a systematic review protocol. JBI Database System Rev Implement Rep 2019;17:1634–67. 10.11124/JBISRIR-2017-004022 31404051

[58] Chen H, Chai Y, Dong L, et al. Effectiveness and appropriateness of mHealth interventions for maternal and child health: systematic review. JMIR Mhealth Uhealth 2018;6:e7. 10.2196/mhealth.8998 29317380 PMC5780618

[59] Watterson JL, Walsh J, Madeka I. Using mHealth to improve usage of antenatal care, postnatal care, and immunization: a systematic review of the literature. Biomed Res Int 2015;2015:153402. 10.1155/2015/153402 26380263 PMC4561933

[60] Sondaal SFV, Browne JL, Amoakoh-Coleman M, et al. Assessing the effect of mHealth interventions in improving maternal and neonatal care in low- and middle-income countries: a systematic review. PLoS One 2016;11:e0154664. 10.1371/journal.pone.0154664 27144393 PMC4856298

[61] Nurmatov UB, Lee SH, Nwaru BI, et al. The effectiveness of mHealth interventions for maternal, newborn and child health in low- and middle-income countries: protocol for a systematic review and meta-analysis. J Glob Health 2014;4:010407. 10.7189/jogh.04.010407 24976966 PMC4073240

[62] Lee SH, Nurmatov UB, Nwaru BI, et al. Effectiveness of mHealth interventions for maternal, newborn and child health in low- and middle-income countries: systematic review and meta-analysis. J Glob Health 2016;6:010401. 10.7189/jogh.06.010401 26649177 PMC4643860

[63] World Health Organization. WHO guideline: recommendations on Digital interventions for health system strengthening. Geneva World Health Organization; 2019. Available: https://www.who.int/publications/i/item/who-digital-implementation-investment-guide 31162915

[64] Haddad SM, Souza RT, Cecatti JG, et al. Correction: building a digital tool for the adoption of the world health organization's antenatal care recommendations: methodological intersection of evidence, clinical logic, and digital technology. J Med Internet Res 2020;22:e24891. 10.2196/24891 33001032 PMC7983224

[65] Patton MQ. Qualitative evaluation checklist: evaluation checklist project. 2013. Available: https://wmich.edu/sites/default/files/attachments/u350/2014/qualitativeevalchecklist.pdf

[66] Suandi D, Williams P, Bhattacharya S. Does involving male partners in antenatal care improve healthcare utilisation? Systematic review and meta-analysis of the published literature from low- and middle-income countries. Int Health 2020;12:484–98. 10.1093/inthealth/ihz073 31613327 PMC11701106

[67] Pope C, Ziebland S, Mays N. Qualitative research in health care. Analysing qualitative data. BMJ 2000;320:114–6. 10.1136/bmj.320.7227.114 10625273 PMC1117368

[68] ATLAS.ti Scientific Software Development GmbH. ATLAS.Ti 23.2.1. ATLAS.Ti scientific software development GmbH; 2023.

