# Supplementary material for: Exploring perceptions and operational considerations for use of a smartphone application to self-monitor blood pressure in pregnancy in Lombok, Indonesia: protocol for a qualitative study
Source: BMJ Open. 2023 Dec 18;13(12):e073875. doi: 10.1136/bmjopen-2023-073875 (PMC10748960; doi:10.1136/bmjopen-2023-073875)
Supplement: Author's manuscript [file bmjopen-2023-073875.draft_revisions.pdf]

BMJ Open

BMJ Open is committed to open peer review. As part of this commitment we make the peer review history of every article we publish publicly available.

When an article is published we post the peer reviewers' comments and the authors' responses online. We also post the versions of the paper that were used during peer review. These are the versions that the peer review comments apply to.

The versions of the paper that follow are the versions that were submitted during the peer review process. They are not the versions of record or the final published versions. They should not be cited or distributed as the published version of this manuscript.

BMJ Open is an open access journal and the full, final, typeset and author-corrected version of record of the manuscript is available on our site with no access controls, subscription charges or pay-per-view fees (http://bmjopen.bmj.com).

If you have any questions on BMJ Open's open peer review process please email info.bmjopen@bmj.com

BMJ Open

Exploring perceptions, barriers and facilitators for the implementation of self-monitoring of blood pressure in pregnancy using a smartphone application in Lombok, Indonesia: Protocol for a qualitative study

Journal:	*BMJ Open*
Manuscript ID	bmjopen-2023-073875
Article Type:	Protocol
Date Submitted by the Author:	21-Mar-2023
Complete List of Authors:	Tamrat, Tigest ; World Health Organization, Department of Sexual and Reproductive Health and Research; University of Geneva<br>Setyawati, Yuni Dwi; Summit Institute for Development<br>Barreix, Maria; World Health Organization<br>Gayatri, Mergy; Summit Institute for Development; Brawijaya University<br>Rinjani, Shannia Oktaviana; Summit Institute for Development<br>Pasaribu, Melissa Paulina; Summit Institute for Development<br>Geissbuhler, Antoine; University of Geneva, HI5lab, Campus Biotech<br>Shankar, Anuraj H; Summit Institute for Development; Oxford University Clinical Research Unit<br>Tunçalp, Özge; World Health Organization, Reproductive Health and Research
Keywords:	Blood Pressure, Information technology < BIOTECHNOLOGY & BIOINFORMATICS, Maternal medicine < OBSTETRICS, Prenatal diagnosis < OBSTETRICS, QUALITATIVE RESEARCH, Self Care

SCHOLARONE™
Manuscripts

Title: Exploring perceptions, barriers and facilitators for the implementation of self-monitoring of blood pressure in pregnancy using a smartphone application in Lombok, Indonesia: Protocol for a qualitative study

Authors:
Tigest Tamrat, [1, 2] Yuni Dwi Setyawati,[3] Maria Barreix[1], Mergy Gayatri[3,4], Shannia Oktaviana Rinjani[3], Melissa Paulina Pasaribu,[3] Antoine Geissbühler,[5] Anuraj H Shankar[3,6*], Ozge Tuncalp[1*]

[1] UNDP/UNFPA/UNICEF/World Bank Special Program of Research, Development and Research Training in Human Reproduction (HRP), Department of Sexual and Reproductive Health and Research, World Health Organization, Geneva, Switzerland

[2] Institute of Global Health, University of Geneva, Geneva, Switzerland

[3]Summit Institute for Development, Mataram, Indonesia

[4]Brawijaya University, Malang, Indonesia

[5]Division of eHealth and Telemedicine, Geneva University Hospitals, Geneva, Switzerland

[6]Oxford University Clinical Research Unit – Indonesia, Jakarta, Indonesia

*Shared senior authorship

Correspondence to: Tigest Tamrat (email: tamratt@who.int)

For peer review only

Abstract

Introduction: Hypertensive disorders of pregnancy (HDP) are a leading cause of maternal deaths globally and require close monitoring of blood pressure (BP) to mitigate potential adverse effects. Despite the recognized need for research on self-monitoring of blood pressure (SMBP) among pregnant populations in low resource settings, there remains a dearth of studies on this topic. The study aims to understand the perceptions, barriers, facilitators, and operational considerations for SMBP by pregnant women using a smartphone-based BP measurement software application in Lombok, Indonesia.

Methods and analysis: This study includes a combination of focus group discussions, in-depth interview, and workshop observations. Pregnant women will also be provided with a research version of the smartphone BP application to use in their home and subsequently provide feedback on their experiences. The study will include pregnant women with current or past hypertensive disorders of pregnancy, their partners, and the health workers involved in the provision and management of antenatal care (ANC) services within the catchment area of six primary health care centers. Data obtained from the interviews and observations will undergo thematic analyses using a combination of both inductive and deductive approaches.

Ethics and dissemination: The study was approved by the WHO/HRP Research Project Review Panel and WHO Ethical Review Committee (A65932) as well as the Health Research Ethics Committee, Faculty of Medicine, Universitas Mataram in Indonesia (004/UN18/F7/ETIK/2023).

Findings will be disseminated through research publications and communicated to the Lombok district health offices. The analyses from this study will also inform the design of a subsequent impact evaluation.

Strengths and Limitations:

- This study addresses a research gap identified by the World Health Organization and expands on the geographic scope of available evidence on SMBP beyond high-income settings.
- The study also uses a novel approach of smartphone-based BP measurement and provides a confluence of digital, maternal health, self-care, and health system considerations, in which qualitative methods will be applied to better understand all these different aspects and how they interact.
- To provide a holistic perspective on the barriers, facilitators and operational requirements for SMBP, the study will include a diverse set of stakeholders of pregnant women, their partners, and health workforce across different levels of the health system.
- This study will inform subsequent impact evaluations and compliments existing initiatives within the research site on the use of digital tools towards identification and management of HDP.
- A limitation of this study is that pregnant women will not be actively self-monitoring their BP to detect clinical changes but rather undergo the process to understand the perceptions of SMBP and implementation considerations.

For peer review only - http://bmjopen.bmj.com/site/about/guidelines.xhtml

Introduction

Hypertensive disorders of pregnancy (HDPs) are one of the most common pregnancy complications(1), affecting approximately 10 percent of all pregnancies(1-4) and resulting in the second highest direct cause of maternal deaths globally (5). HDPs can be broadly classified as chronic hypertension, gestational hypertension, and preeclampsia-eclampsia.(1, 3) Chronic hypertension consists of elevated blood pressure (BP) that is identified prior to the pregnancy or detected within the first 20 weeks. (1) Gestational hypertension is considered to be induced during a pregnancy with the onset of elevated BP after 20 weeks. (1) Pre-eclampsia is the development of gestational hypertension combined with presence of proteinuria and is a precursor to eclampsia, a life-threatening condition which accounts for the majority of deaths attributed to HDPs.(2, 6)

The World Health Organization (WHO) recommends routine measurement of BP during pregnancy and at every point of contact in the provision of antenatal care (ANC) as part of efforts to improve maternal health.(7, 8) In certain contexts, pregnant women identified with HDP may require additional follow-up and monitoring in order to mitigate potential adverse outcomes.(9) Furthermore, as the underlying cause of pre-eclampsia is unknown and onset may be triggered in between clinical consultations, close monitoring of BP beyond the health facility is critical. (3, 10, 11)

Self-monitoring of blood pressure

Self-monitoring of blood pressure (SMBP), also known as home-based BP monitoring, is well researched in the general population (12-17) with a WHO recommendation in 2013 supporting its appropriate and affordable use.(18) Cited advantages include improved BP control and reduced anxiety associated with 'white coat' hypertension. (10-12, 15, 17) However, studies also highlight challenges associated with the variety of devices used within home settings, reliability of readings and calibration requirements, as well as ensuring guidance to individuals on interpreting results, dealing with variations of readings, and appropriate actions to be taken. (19, 20)

SMBP during pregnancy is an emerging approach recommended by WHO in 2021 "as an additional option to clinic blood pressure monitoring by health workers during antenatal contacts only, for individuals with hypertensive disorders of pregnancy."(21) Although there is a substantial literature base on SMBP in non-pregnant populations (12-16, 19, 20, 22-28), the evidence specific to pregnancy is still nascent (11, 29-32) and limited for low- and middle-income countries (LMIC) where there remains a high burden of HDPs.(33) To date, studies that explore SMBP among pregnant women span Australia, Canada, Japan, United Kingdom (UK), New Zealand, and United States (US) (34, 35), with one study in South Africa focused on knowledge and attitudes of SMBP among preeclamptic patients.(33) A Cochrane systematic review on the different approaches to SMBP in pregnancy indicated uncertainties in the benefits of SMBP of hypertensive pregnancies due to limited number of studies.(36) Other systematic reviews on SMBP during antenatal and postpartum periods demonstrated its feasibility but no difference in clinical outcomes (37), while another metanalyses reported reductions in antenatal care visits, hospital admissions, and diagnosis of preeclampsia (38). All systematic reviews highlighted the lack of included studies from LMICs and the need for more evidence on this topic. (36-38)

Among the available studies, the Blood Pressure Self-Monitoring in Pregnancy (BuMP) research initiative conducted in the UK, offers one of the most comprehensive methods for evaluating feasibility and effectiveness. (29, 30, 39-41) For the feasibility assessment, women between 12-16 weeks gestational age and identified to be at risk for pre-eclampsia were recruited to self-monitor their BP using an automated BP monitor (Microlife WatchBP Home validated for use in pregnancy and pre-eclampsia). Study participants had the option to also text their readings and receive automated responses and text prompts. (30) Women reported that the approach was reassuring and made them more informed about

For peer review only - http://bmjopen.bmj.com/site/about/guidelines.xhtml

HDPs; however, study authors noted the limited generalizability of the findings across different socioeconomic backgrounds.(30) This assessment was followed by a prospective cohort study of approximately 200 women, demonstrating that women at risk for pre-eclampsia were able to obtain more frequent BP readings and facilitate the detection of gestational hypertension in-between clinic visits.(29) This study also explored healthcare providers' perspectives and noted their concerns in reconciling the autonomy and reassurance that women may gain in exchange with the caution and uncertainty that self-monitoring requires.(40) More recently, the BuMP study conducted a randomized clinical trial (RCT) among 2441 pregnant individuals at increased risk for preeclampsia, in which there was no statistically significant differences in early detection of preeclampsia between SMBP and standard care groups (42). Similarly, there was also no statistically significant differences among pregnant individuals with chronic or gestational hypertension.(43)

Another RCT, also conducted in the UK, enrolled pregnant women with chronic hypertension or gestational hypertension for SMBP using a calibrated BP measurement device (Microlife WatchBP Home); women with pre-eclampsia were excluded from this study. (44) Study participants reported their second of two BP readings via text or through an app designed for the study or noted it in on paper to be reviewed at their ANC contact.(44) In response to the mobile phone submission, participants received immediate automated responses. Although there was no difference in the mean systolic BP measurements or medication adherence, participants demonstrated willingness and acceptability for self-monitoring.(44)

Potential for digital technology to facilitate SMBP

Advances in digital technology in LMICs, primarily through penetration of mobile devices(45), provide an opportunity to expand SMBP and other health monitoring services. While the catalyst for SBPM in high income settings was spurred by the rise of automated cuffs(12), this has not gained traction in settings in which there is already a limited availability of automated equipment for facility based use.(46) Furthermore, BP measurement devices require additional validation specific to pregnant populations.(47, 48)

As an emerging alternative to measuring BP, OptiBP™ is a software application that utilizes smartphone cameras to capture optical pulse-waves of the fingertip and estimate BP readings (Figure 1). The OptiBP™ application runs on Android OS 8.1 and leverages the smartphone camera to record photoplethysmographic (PPG) optical pulse waves derived from blood volume changes at the fingertips and estimate BP values using an algorithm.(49, 50). The OptiBP™ application has undergone validation in Switzerland in which it demonstrated concordance against reference BP measurements; (50, 51) additional validation studies towards regulatory approval are in progress. [cite] To also ensure the accuracy of OptiBP across different environmental conditions and demographic profiles, the WHO conducted a multi-site validation in accordance with the ISO regulatory standards Tanzania, South Africa, and Bangladesh.(52) This study included a targeted assessment on the accuracy of BP measurement among pregnant populations to account for the maternal cardiovascular changes on the detection of optical pulse waves.(53) A similar validation study was also conducted in Indonesia among pregnant populations in West Lombok and East Lombok in West Nusa Tenggara Province. This was complimented by a research study to integrate OptiBP™ into the decision support tools that health workers use for antenatal care as an approach to incorporating into routine service delivery.

Figure 1 Optical pulse wave technology underlying OptiBP smartphone application(50)

For peer review only

Aims and Objectives

The newfound capability of using a smartphone application for BP measurement presents an opportunity for broadening SMBP to LMIC settings, where coverage of health services is more limited and mobile technologies have gained widespread use for maternal health.(53-62) Using these advances in BP monitoring, this research addresses evidence gaps identified in the WHO guideline on self-care interventions regarding the feasibility, benefits, and harms of SMBP among individuals with HDP.(21) This study aims to understand the perceptions, barriers, and operational considerations for SMBP via a smartphone application (OptiBP) in primary health care settings of Lombok, Indonesia. The first objective of this study is to explore pregnant women's, their partners, and health workers' attitudes towards SMBP in general, and specifically regarding the use of a smartphone application for SMBP. In addition, the study will include health system managers and examine the implementation requirements through user-centered design approaches to adapt for health literacy and workflow integration requirements.

Methods and analysis:

Study design and setting

This study will use a combination of qualitative methods, including focus group discussions (FGD), in-depth interview (IDI), and workshop observations to triangulate findings on perceptions and operational considerations for SMBP by pregnant women. The study will include pregnant women, their partners, and the health workers involved in the provision and management of ANC services to obtain a holistic understanding of the dynamics across health system actors. To derive concrete operational considerations, selected pregnant women will also be provided with a research version of the OptiBP™ application to use in their home setting and subsequently provide feedback on their experiences. The study will be conducted within the catchment area of six primary health care facilities in West Lombok district Lombok, Indonesia. Data collection is started in mid-February 2023 and expected to be completed by May 2023.

Participant selection

The main population for this study is pregnant women with current or past hypertensive disorders of pregnancy. Eligible participants will be drawn from those pregnant women coming to receive ANC at the selected study facilities and registered in the ANC digital module, which is a digital health record tracking and decision support system that was implemented independently of this study.(63, 64) Following approval of requests from the heads of health facilities to review the digital health records for sampling purposes, the study team will select pregnant women for screening and invitation to participate in the study. A subset of partners of the pregnant women included in the study will also be selected following the consent of the pregnant woman to contact her partner.

A diverse range of health workforce cadres, including community health workers, community and facility-based midwives, obstetrician/gynecologists, and health system managers will also be included to compliment the findings from pregnant women. Table 1 provides an overview of the participants and sampling frame.

Table 1. Sampling frame

Activity/Method	Pregnant women with HDP	Partners of pregnant	Health workers/ manager

	<30 years	>=30 years	**women**	CHWs	Nurse-midwives (community)	Nurse-midwives (facility)	Doctors	Health system managers
FGD on perception of SMBP	2-3 FGDs of 5-7	2-3 FGDs of 5-7	2 FGDs of 5-7	4 FGDs of 5-7	2-3 FGDs of 5-7	2-3 FGDs of 5-7	N/A	N/A
IDI on perception of SMBP	4-5	4-5	N/A	4-5	4-5	4-5	3-5	3-5
Intervention adaptation workshops	5-10	5-10	N/A	N/A	N/A	N/A	N/A	N/A
FGD: Focus Group Discussion; IDI: In-depth Interview; HDP: Hypertensive diseases in pregnancy; N/A: Not applicable								

Sampling and recruitment

The study will use purposive sampling of pregnant women with a current diagnosis or history of HDP (as derived from their medical record in the ANC digital module) and across two age groups to account for potentially differing levels of digital literacy. We will identify women with different educational levels, gestational age, and parity through the sampling and collect variables on these factors.

Pregnant women will be approached during their routine ANC contacts once they have completed their consultation. A research assistant will brief women on the study and gauge their interest to participate. The contact information of consenting and eligible pregnant women will be taken, and the research assistant will inform them that they will be contacted if they are selected for the study. Eligibility for pregnant participants will be based on the following criteria: confirmed pregnancy (either by health worker or self-administered and documented rapid test), current or history of HDP (derived from the digital record system), greater than 18 years of age or emancipated minor, living within catchment area of selected health facilities, able to read one sentence in Bahasa, and having given informed consent. Pregnant women will be excluded if they have severe visual impairment that prevents use of the smartphone application (e.g., blindness) or absence of index or middle finger on either left or right hand. Partners of pregnant women will be identified through the recruited women after first obtaining consent from the pregnant women to contact their partners.

Healthcare managers will be selected based on their role and oversight in the catchment area. They will be invited to participate in the study and asked for their informed consent during their work hours. The different cadres of health workers will be identified through District Health Office (DHO) and facility networks under its hierarchical systems, which are Public Health Centers (Puskesmas) and Maternity Clinics (Polindes).

Health workers (nurse-midwives and community health workers) will be randomly selected to participate in the study based on a list provided by the DHO. The study team will first briefly inform by the supervisors and invite the selected health workers to attend an information session where the research staff will elaborate further on the study and ask for their consent to take part in the study. The interviews will take place during work hours after permission has been obtained from the DHO.

The study will invite women to either be part of the FGDs or workshops and randomly allocate to the two groups. Participants for the IDIs will be sampled from the FGDs to examine their feedback in greater detail. The sample size for pregnant women and health workers will be based on requirements for

For peer review only

reaching saturation, as well as using an emergent sampling design, in which participants will be recruited as the study progresses.(65)

Methods of approach

Interviews

FGDs and IDIs will be used to explore potential barriers and motivators for SMBP, as well as how this may influence changes in pregnant women's interactions with health workers. As partners and family members of pregnant women can influence access and use of ANC services, their perceptions of SMBP will also be used to obtain a comprehensive understanding of the considerations for SMBP and any related care.(66)

Observation workshops

In addition to the FGDs and IDIs, observation workshops will be held to review the smartphone application interface and determine adaptation requirements for enhancing usability in accordance with health literacy levels and ensuring integration with health system and data workflows. The workshop will be conducted in two parts. First, we will introduce a research version of the smartphone application to selected women and observe initial reactions and feedback. This version of the application would be linked to a de-identified study account and include a disclaimer stating the measurements are not to be used for clinical decision-making and that participants should contact health facility and research team if they face any health issues. The application will not be used by the research team or health workers for any clinical decision making, and all BP measurements for service provision will be done through the standard care at facilities using standard sphygmomanometers. Women will be provided with a smartphone with the application and instructions to explore using it at home twice a day for one week before returning to the subsequent workshop, one week later. This second workshop will be used to gather feedback on their experiences of use and refine guidance/training support needed for achieving fidelity and requirements for integrating within health system workflows.

Both workshops will include structured and unstructured observations of pregnant women engaging with the smartphone application interface and will be used to further inform the decision support prompts (e.g., what to do in cases of high BP reading) that are contextually appropriate and resonate with the levels of health literacy. In the structured observations, pregnant women will be asked to interact with the smartphone application prototype across various tasks and scenarios, such as turning on the smartphone application, placing their finger on the camera of the smartphone for taking their BP measurement, interpreting the result/output from the BP measurement, and undertaking follow-up actions, when necessary.

Data collection

For the interviews, a semi-structured topic guide will be provided to trained interviewers conducting the FGDs and IDIs. The topic guide for pregnant individuals will cover areas of pregnancy experience, mobile phone use in pregnancy, knowledge of and experience with taking BP, self-efficacy in taking BP, trust and support for taking BP. Interview guides for the health worker will include topics surrounding experience in providing ANC, perceptions of self-management of pregnancy generally, perceptions of SMBP by pregnant women, and data flow and health system linkages. All interview guides will include probes and questions for initiating the interviews and allow for open-ended responses and capturing any reflections that may emerge. Interviews will be recorded, transcribed and translated into English for analysis.

For the observation workshops, a research assistant will document both unprompted reactions, as well as responses to specific tasks with the smartphone application. As a supplement to the observation notes,

For peer review only

study will also embed a screen recorder while pregnant women use the application during the workshop to capture the process flows and interactions with the application, including length of time and frequency of use of different buttons. The study team will also take videos and photos during the observation workshop to record feedback related to interactions with the application.

Analysis

Recordings will be double transcribed and translated for quality assurance. Data obtained from the interviews and observations will first undergo an initial rapid analysis to flag key themes and considerations, including findings from the existing literature base. This will be followed by detailed analysis and coding using a thematic analysis employing a combination of both inductive and deductive approaches. (65, 67) Responses will first be reviewed for familiarization of themes and then be coded emergent categories.(67) Each finding will be compared and crosschecked to determine whether it aligns with existing categories or if a new theme should be developed.(65) The analysis will be conducted by two individuals to ensure interrater reliability and then reviewed collectively with the broader research team. Screen recordings of interactions with the application will also be reviewed to identify patterns of use and Atlas.ti will be used to manage the coding and analysis of themes.

Patient and public involvement

The views of patients or the public will be reflected in the analysis of the manuscript and through the co-design workshops of the study.

Ethics and dissemination:

The study was approved by the WHO/HRP Research Review Panel and WHO Ethical Review Committee (Reference number A65932) as well as the Health Research Ethics Committee, Faculty of Medicine, Universitas Mataram in Indonesia (004/UN18/F7/ETIK/2023).

All eligible participants will be provided with an information sheet and required to provide informed written consent before participation. Confidential data will be stored at country level and de-identified transcriptions will be shared with WHO to support the analyses. Personal identifiers will be removed during the transcription of audio recordings and labelled with a de-identified participant identification number. Identifiable data will be kept at the research site in a secure location for up to two years.

Participants using the smartphone application at home will be provided with detailed guidance, as well as airtime/SIM cards to contact the research team as needed. The smartphone application will not be used by the research team or health workers for any clinical decision making, and all BP measurements for service provision will be done through the standard care at facilities using standard sphygmomanometers.

Photos and video recordings will only be taken during trainings and specific group events, and only after informed consent. Unless otherwise consented, use of these will be obscured by blurring or other effects to render persons non-identifiable, and the purpose for use will be restricted to training and implementing processes to make the work or intervention more effective. The company that developed the smartphone application will not have access to any data or transcripts but will have access to the feedback shared during their participation in the workshop.

Findings will be disseminated through research publications and communicated to the DHO. The analyses from this study will also inform the design of a subsequent impact evaluation. In addition, the feedback from workshops may contribute to potential refinements to the smartphone application.

Authors' contributions: TT, YS, MB, AS, and OT developed the protocol, from which excerpts were used to draft this manuscript. AG and MG provided comments on both the protocol and manuscript. TT prepared the first draft of the manuscript. All authors reviewed both the protocol and this manuscript. AT and AS are shared senior authors.

Acknowledgements: We thank the heads of health centers from the study sites (Penimbung, Ranjok, Jembatan Kembar, Narmada Gerung, and Meninting) support to conduct the study. We are also grateful to Dr Garrett Mehl (WHO) for support in conceptualization of the study.

Funding: This work was funded by the Bill and Melinda Gates Foundation (grant number OPP1201339) and the UNDP-UNFPA-UNICEF-WHO-World Bank Special Programme of Research, Development and Research Training in Human Reproduction (HRP), a cosponsored programme executed by the World Health Organization (WHO). This research received no specific grant from any funding agency in the public, commercial or not-for-profit sectors.

Competing interests: None declared from authors. Biospectal, the company that developed the OptiBP application, will provide a research version of the application for use in the study but did not play a role in the study design.

Disclaimer: The authors alone are responsible for the views expressed in this article and they do not necessarily represent the views, decisions, or policies of the institutions with which they are affiliated.

References:

1. Mammaro A, Carrara S, Cavaliere A, Ermito S, Dinatale A, Pappalardo EM, et al. Hypertensive disorders of pregnancy. J Prenat Med. 2009;3(1):1-5.
2. Duley L. The global impact of pre-eclampsia and eclampsia. Semin Perinatol. 2009;33(3):130-7.
3. WHO Recommendations for Prevention and Treatment of Pre-Eclampsia and Eclampsia. WHO Guidelines Approved by the Guidelines Review Committee. Geneva2011.
4. Steegers EA, von Dadelszen P, Duvekot JJ, Pijnenborg R. Pre-eclampsia. Lancet. 2010;376(9741):631-44.
5. Say L, Chou D, Gemmill A, Tuncalp O, Moller AB, Daniels J, et al. Global causes of maternal death: a WHO systematic analysis. Lancet Glob Health. 2014;2(6):e323-33.
6. Abalos E, Cuesta C, Grosso AL, Chou D, Say L. Global and regional estimates of preeclampsia and eclampsia: a systematic review. Eur J Obstet Gynecol Reprod Biol. 2013;170(1):1-7.
7. WHO Recommendations on Antenatal Care for a Positive Pregnancy Experience. WHO Guidelines Approved by the Guidelines Review Committee. Geneva2016.
8. Tsakane MAG Hlongwane BB, Maria C Barreix, Robert Pattinson, Metin Gülmezoglu, Valerie Vannevel , Özge Tunçalp. Implementing antenatal care recommendations, South Africa. Bulletin of the World Health Organization. 2021;99::220-7.
9. Excellence NIfHaC. Hypertension in pregnancy: diagnosis and management -- NICE guideline [NG133] United Kingdom2019 [Available from: https://www.nice.org.uk/guidance/ng133/chapter/Recommendations.
10. Mustafa R, Ahmed S, Gupta A, Venuto RC. A comprehensive review of hypertension in pregnancy. J Pregnancy. 2012;2012:105918.
11. Hodgkinson JA, Tucker KL, Crawford C, Greenfield SM, Heneghan C, Hinton L, et al. Is self monitoring of blood pressure in pregnancy safe and effective? BMJ. 2014;349:g6616.
12. Parati G, Stergiou GS, Asmar R, Bilo G, de Leeuw P, Imai Y, et al. European Society of Hypertension practice guidelines for home blood pressure monitoring. J Hum Hypertens. 2010;24(12):779-85.

For peer review only

13. Imai Y, Obara T, Asamaya K, Ohkubo T. The reason why home blood pressure measurements are preferred over clinic or ambulatory blood pressure in Japan. Hypertens Res. 2013;36(8):661-72.
14. Roerecke M, Kaczorowski J, Myers MG. Comparing Automated Office Blood Pressure Readings With Other Methods of Blood Pressure Measurement for Identifying Patients With Possible Hypertension: A Systematic Review and Meta-analysis. (2168-6114 (Electronic)).
15. Bo Y, Kwok KO, Chu KK, Leung EY, Yu CP, Wong SY, et al. Comparison Between Automated Office Blood Pressure Measurements and Manual Office Blood Pressure Measurements-Implications in Individual Patients: a Systematic Review and Meta-analysis. (1534-3111 (Electronic)).
16. Niiranen TJ, Jula Am Fau - Kantola IM, Kantola Im Fau - Reunanen A, Reunanen A. Comparison of agreement between clinic and home-measured blood pressure in the Finnish population: the Finn-HOME Study. (0263-6352 (Print)).
17. McManus RJ, Little P, Stuart B, Morton K, Raftery J, Kelly J, et al. Home and Online Management and Evaluation of Blood Pressure (HOME BP) using a digital intervention in poorly controlled hypertension: randomised controlled trial. Bmj. 2021;372:m4858.
18. World Health Organization. Package of Essential Noncommunicable (PEN) disease interventions for primary health care in low-resource settings. Geneva, Switzerland2013.
19. Fletcher BR, Hinton L, Hartmann-Boyce J, Roberts NW, Bobrovitz N, McManus RJ. Self-monitoring blood pressure in hypertension, patient and provider perspectives: A systematic review and thematic synthesis. Patient education and counseling. 2016;99(2):210-9.
20. McManus RJ, Ryan A, Greenfield S, Pattison HM, Clifford S, Marriott J, et al. Self measurement of blood pressure: a community survey. Journal of Human Hypertension. 2007;21(9):741-3.
21. World Health Organization. WHO Consolidated Guideline on Self-Care Interventions for Health v.2 Sexual and Reproductive Health and Rights Geneva: World Health Organization; 2022 [Available from: https://www.who.int/publications/i/item/9789240052192.
22. Monahan M, Jowett S, Lovibond K, Gill P, Godwin M, Greenfield S, et al. Predicting Out-of-Office Blood Pressure in the Clinic for the Diagnosis of Hypertension in Primary Care: An Economic Evaluation. Hypertension. 2018;71(2):250-61.
23. Hodgkinson J, Mant J, Martin U, Guo B, Hobbs FD, Deeks JJ, et al. Relative effectiveness of clinic and home blood pressure monitoring compared with ambulatory blood pressure monitoring in diagnosis of hypertension: systematic review. BMJ. 2011;342:d3621.
24. Beyhaghi H, Viera AJ. Comparative Cost-Effectiveness of Clinic, Home, or Ambulatory Blood Pressure Measurement for Hypertension Diagnosis in US Adults. Hypertension. 2019;73(1):121-31.
25. Tucker K, Sheppard JP, Stevens R, Bosworth HB, Bove A, Bray EP, et al. [OP.6B.04] INDIVIDUAL PATIENT DATA META-ANALYSIS OF SELF-MONITORING OF BLOOD PRESSURE (BP-SMART). Journal of Hypertension. 2016;34.
26. Bray EP, Holder R, Mant J, McManus RJ. Does self-monitoring reduce blood pressure? Meta-analysis with meta-regression of randomized controlled trials. Database of Abstracts of Reviews of Effects (DARE). 2011.
27. Uhlig K, Patel K, Ip S, Kitsios GD, Balk EM. Self-Measured Blood Pressure Monitoring in the Management of Hypertension: A Systematic Review and Meta-analysis. Annals of internal medicine. 2013;159(3):185-94.
28. Ward AM, Takahashi O, Stevens R, Heneghan C. Home measurement of blood pressure and cardiovascular disease: systematic review and meta-analysis of prospective studies. J Hypertens. 2012;30(3):449-56.
29. Tucker KL, Taylor KS, Crawford C, Hodgkinson JA, Bankhead C, Carver T, et al. Blood pressure self-monitoring in pregnancy: examining feasibility in a prospective cohort study. BMC Pregnancy Childbirth. 2017;17(1):442.

For peer review only

30. Hinton L, Tucker KL, Greenfield SM, Hodgkinson JA, Mackillop L, McCourt C, et al. Blood pressure self-monitoring in pregnancy (BuMP) feasibility study; a qualitative analysis of women's experiences of self-monitoring. BMC Pregnancy Childbirth. 2017;17(1):427.
31. Tucker KL, Bowen L, Crawford C, Mallon P, Hinton L, Lee MM, et al. The feasibility and acceptability of self-testing for proteinuria during pregnancy: A mixed methods approach. Pregnancy Hypertens. 2018;12:161-8.
32. Wilson H, Tucker KL, Chisholm A, Hodgkinson J, Lavallee L, Mackillop L, et al. Self-monitoring of blood pressure in pregnancy: A mixed methods evaluation of a national roll-out in the context of a pandemic. Pregnancy Hypertension. 2022;30:7-12.
33. Munyungula J, Shakwane S. Self-monitoring of blood pressure for preeclampsia patients: Knowledge and attitudes. Curationis. 2021;44(1):e1-e8.
34. Tucker KL, Bankhead C, Hodgkinson J, Roberts N, Stevens R, Heneghan C, et al. How Do Home and Clinic Blood Pressure Readings Compare in Pregnancy? Hypertension. 2018;72(3):686-94.
35. Aquino M, Munce S, Griffith J, Pakosh M, Munnery M, Seto E. Exploring the Use of Telemonitoring for Patients at High Risk for Hypertensive Disorders of Pregnancy in the Antepartum and Postpartum Periods: Scoping Review. JMIR Mhealth Uhealth. 2020;8(4):e15095.
36. Ashworth DC, Maule SP, Stewart F, Nathan HL, Shennan AH, Chappell LC. Setting and techniques for monitoring blood pressure during pregnancy. Cochrane Database Syst Rev. 2020;8(8):Cd012739.
37. Yeh PT, Rhee DK, Kennedy CE, Zera CA, Lucido B, Tuncalp O, et al. Self-monitoring of blood pressure among women with hypertensive disorders of pregnancy: a systematic review. BMC Pregnancy Childbirth. 2022;22(1):454.
38. Kalafat E, Benlioglu C, Thilaganathan B, Khalil A. Home blood pressure monitoring in the antenatal and postpartum period: A systematic review meta-analysis. Pregnancy Hypertens. 2020;19:44-51.
39. Dougall G, Franssen M, Tucker KL, Yu LM, Hinton L, Rivero-Arias O, et al. Blood pressure monitoring in high-risk pregnancy to improve the detection and monitoring of hypertension (the BUMP 1 and 2 trials): protocol for two linked randomised controlled trials. BMJ Open. 2020;10(1):e034593.
40. Hinton L, Hodgkinson J, Tucker KL, Rozmovits L, Chappell L, Greenfield S, et al. Exploring the potential for introducing home monitoring of blood pressure during pregnancy into maternity care: current views and experiences of staff-a qualitative study. BMJ Open. 2020;10(12):e037874.
41. Dougall G, Franssen M, Tucker KL, Yu L-M, Hinton L, Rivero-Arias O, et al. Blood pressure monitoring in high-risk pregnancy to improve the detection and monitoring of hypertension (the BUMP 1 and 2 trials): protocol for two linked randomised controlled trials. BMJ Open. 2020;10(1):e034593.
42. Tucker KL, Mort S, Yu LM, Campbell H, Rivero-Arias O, Wilson HM, et al. Effect of Self-monitoring of Blood Pressure on Diagnosis of Hypertension During Higher-Risk Pregnancy: The BUMP 1 Randomized Clinical Trial. Jama. 2022;327(17):1656-65.
43. Chappell LC, Tucker KL, Galal U, Yu LM, Campbell H, Rivero-Arias O, et al. Effect of Self-monitoring of Blood Pressure on Blood Pressure Control in Pregnant Individuals With Chronic or Gestational Hypertension: The BUMP 2 Randomized Clinical Trial. Jama. 2022;327(17):1666-78.
44. Pealing LM, Tucker KL, Mackillop LH, Crawford C, Wilson H, Nickless A, et al. A randomised controlled trial of blood pressure self-monitoring in the management of hypertensive pregnancy. OPTIMUM-BP: A feasibility trial. Pregnancy Hypertens. 2019;18:141-9.
45. International Telecommunications Union. ICT Facts and Figures. Geneva: 2015 (Avaliable from https://www.itu.int/en/ITU-D/Statistics/Documents/facts/ICTFactsFigures2015.pdf, accessed 23 March 2018)
46. Betran AP, Bergel E, Griffin S, Melo A, Nguyen MH, Carbonell A, et al. Provision of medical supply kits to improve quality of antenatal care in Mozambique: a stepped-wedge cluster randomised trial. Lancet Glob Health. 2018;6(1):e57-e65.

47. Ashworth DC, Maule SP, Stewart F, Nathan HL, Shennan AH, Chappell LC. Setting and techniques for monitoring blood pressure during pregnancy. Cochrane Database Syst Rev. 2020;8:CD012739.
48. O'Brien E, Atkins N, Stergiou G, Karpettas N, Parati G, Asmar R, et al. European Society of Hypertension International Protocol revision 2010 for the validation of blood pressure measuring devices in adults. Blood Pressure Monitoring. 2010;15(1):23-38.
49. Ghamri Y, Proença M, Hofmann G, Renevey P, Bonnier G, Braun F, et al. Automated Pulse Oximeter Waveform Analysis to Track Changes in Blood Pressure During Anesthesia Induction: A Proof-of-Concept Study. (1526-7598 (Electronic)).
50. Schoettker P, Degott J, Hofmann G, Proença M, Bonnier G, Lemkaddem A, et al. Blood pressure measurements with the OptiBP smartphone app validated against reference auscultatory measurements. Scientific Reports. 2020;10(1):17827.
51. Degott J, Ghajarzadeh-Wurzner A, Hofmann G, Proença M, Bonnier G, Lemkaddem A, et al. Smartphone based blood pressure measurement: accuracy of the OptiBP mobile application according to the AAMI/ESH/ISO universal validation protocol. Blood Pressure Monitoring. 2021.
52. Tigest Tamrat. Charles Festo VV, Tsakane Hlongwane, Hasmot Ali, Getrud Mollel, Kaniz Fahmida, Kelsey Alland, Maria Barreix, Hedieh Mehrtash, Ronaldo Silva, Soe Soe Thwin, Garrett Mehl, Alain Labrique, Honorati Masanja, and Özge Tunçalp,. Accuracy of a smartphone application for blood pressure estimation in Bangladesh, South Africa, and Tanzania. npj Digital Medicine. 2023 *in press*.
53. Su F, Li Z, Sun X, Han N, Wang L, Luo X. The pulse wave analysis of normal pregnancy: investigating the gestational effects on photoplethysmographic signals. Biomed Mater Eng. 2014;24(1):209-19.
54. Saronga NJ, Burrows T, Collins CE, Ashman AM, Rollo ME. mHealth interventions targeting pregnancy intakes in low and lower-middle income countries: Systematic review. Matern Child Nutr. 2019;15(2):e12777.
55. Ilozumba O, Abejirinde IO, Dieleman M, Bardaji A, Broerse JEW, Van Belle S. Targeting strategies of mHealth interventions for maternal health in low and middle-income countries: a systematic review protocol. BMJ Open. 2018;8(2):e019345.
56. Dol J, Campbell-Yeo M, Tomblin Murphy G, Aston M, McMillan D, Richardson B. Impact of mobile health interventions during the perinatal period for mothers in low- and middle-income countries: a systematic review protocol. JBI Database System Rev Implement Rep. 2019;17(2):137-46.
57. Chen H, Chai Y, Dong L, Niu W, Zhang P. Effectiveness and Appropriateness of mHealth Interventions for Maternal and Child Health: Systematic Review. JMIR Mhealth Uhealth. 2018;6(1):e7.
58. Watterson JL, Walsh J, Madeka I. Using mHealth to Improve Usage of Antenatal Care, Postnatal Care, and Immunization: A Systematic Review of the Literature. Biomed Res Int. 2015;2015:153402.
59. Sondaal SF, Browne JL, Amoakoh-Coleman M, Borgstein A, Miltenburg AS, Verwijs M, et al. Assessing the Effect of mHealth Interventions in Improving Maternal and Neonatal Care in Low- and Middle-Income Countries: A Systematic Review. PLoS One. 2016;11(5):e0154664.
60. Nurmatov UB, Lee SH, Nwaru BI, Mukherjee M, Grant L, Pagliari C. The effectiveness of mHealth interventions for maternal, newborn and child health in low- and middle-income countries: Protocol for a systematic review and meta-analysis. J Glob Health. 2014;4(1):010407.
61. Lee SH, Nurmatov UB, Nwaru BI, Mukherjee M, Grant L, Pagliari C. Effectiveness of mHealth interventions for maternal, newborn and child health in low- and middle-income countries: Systematic review and meta-analysis. J Glob Health. 2016;6(1):010401.
62. World Health Organization. WHO guideline:: Recommendations on digital interventions for health system strengthening Geneva: World Health Organization; 2019 [124]. Available from: https://www.who.int/publications/i/item/who-digital-implementation-investment-guide.
63. Kostanjsek Nea. Use of ICD-11 and other WHO classifications and terminologies in clinical and public health guideline development and implementation.

For peer review only

64. Tamrat T, Chandir S, Alland K, Pedrana A, Shah MT, Footitt C, et al. Digitalization of routine health information systems: Bangladesh, Indonesia, Pakistan. Bull World Health Organ. 2022;100(10):590-600.
65. Patton MQ. Qualitative evaluation checklist: Evaluation Checklist Project; 2013 [Available from: https://wmich.edu/sites/default/files/attachments/u350/2014/qualitativeevalchecklist.pdf.
66. Suandi D, Williams P, Bhattacharya S. Does involving male partners in antenatal care improve healthcare utilisation? Systematic review and meta-analysis of the published literature from low- and middle-income countries. Int Health. 2020;12(5):484-98.
67. Pope C, Ziebland S, Mays N. Qualitative research in health care. Analysing qualitative data. BMJ. 2000;320(7227):114-6.

For peer review only

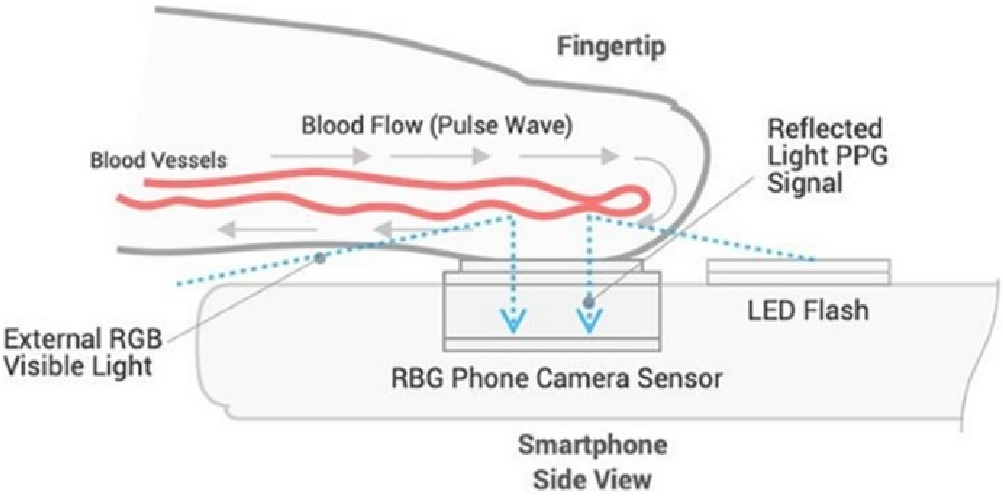

Figure 1 Optical pulse wave technology underlying OptiBP smartphone application(50)

79x39mm (220 x 220 DPI)

BMJ Open

Exploring perceptions, barriers and facilitators for self-monitoring of blood pressure in pregnancy using a smartphone application in Lombok, Indonesia: Protocol for a qualitative study

Journal:	*BMJ Open*
Manuscript ID	bmjopen-2023-073875.R1
Article Type:	Protocol
Date Submitted by the Author:	20-Nov-2023
Complete List of Authors:	Tamrat, Tigest ; World Health Organization, Department of Sexual and Reproductive Health and Research; University of Geneva<br>Setyawati, Yuni Dwi; Summit Institute for Development<br>Barreix, Maria; World Health Organization<br>Gayatri, Mergy; Summit Institute for Development; Brawijaya University<br>Rinjani, Shannia Oktaviana; Summit Institute for Development<br>Pasaribu, Melissa Paulina; Summit Institute for Development<br>Geissbuhler, Antoine; University of Geneva, HI5lab, Faculty of Medicine<br>Shankar, Anuraj H; Summit Institute for Development; Oxford University Clinical Research Unit<br>Tunçalp, Özge; World Health Organization, Reproductive Health and Research
**Primary Subject Heading**:	Public health
Secondary Subject Heading:	Reproductive medicine, Qualitative research, Patient-centred medicine, Obstetrics and gynaecology, Health informatics
Keywords:	Blood Pressure, Information technology < BIOTECHNOLOGY & BIOINFORMATICS, Maternal medicine < OBSTETRICS, Prenatal diagnosis < OBSTETRICS, QUALITATIVE RESEARCH, Self Care

SCHOLARONE™
Manuscripts

Title: Exploring perceptions, barriers, and facilitators for self-monitoring of blood pressure in pregnancy using a smartphone application in Lombok, Indonesia: Protocol for a qualitative study

Authors:
Tigest Tamrat, [1, 2] Yuni Dwi Setyawati,[3] Maria Barreix[1], Mergy Gayatri[3,4], Shannia Oktaviana Rinjani[3], Melissa Paulina Pasaribu,[3] Antoine Geissbühler,[2,5] Anuraj H Shankar[3,6*], Ozge Tuncalp[1*]

[1] UNDP/UNFPA/UNICEF/World Bank Special Program of Research, Development and Research Training in Human Reproduction (HRP), Department of Sexual and Reproductive Health and Research, World Health Organization, Geneva, Switzerland

[2] Institute of Global Health, University of Geneva, Geneva, Switzerland

[3]Summit Institute for Development, Mataram, Indonesia

[4]Brawijaya University, Malang, Indonesia

[5]HI5lab, Faculty of Medicine, University of Geneva, Geneva, Switzerland

[6]Oxford University Clinical Research Unit – Indonesia, Jakarta, Indonesia

*Shared senior authorship

Correspondence to: Tigest Tamrat (email: tamratt@who.int)

For peer review only - http://bmjopen.bmj.com/site/about/guidelines.xhtml

Abstract

Introduction: Hypertensive disorders of pregnancy (HDP) are a leading cause of maternal deaths globally and require close monitoring of blood pressure (BP) to mitigate potential adverse effects. Despite the recognized need for research on self-monitoring of blood pressure (SMBP) among pregnant populations in low resource settings, there remains a dearth of studies on this topic. The study aims to understand the perceptions, barriers, facilitators, and operational considerations for SMBP by pregnant women using a smartphone-based BP measurement software application in Lombok, Indonesia.

Methods and analysis: This study includes a combination of focus group discussions, in-depth interview, and workshop observations. Pregnant women will also be provided with a research version of the smartphone BP application to use in their home and subsequently provide feedback on their experiences. The study will include pregnant women with current or past hypertensive disorders of pregnancy, their partners, and the health workers involved in the provision and management of antenatal care (ANC) services within the catchment area of six primary health care centers. Data obtained from the interviews and observations will undergo thematic analyses using a combination of both inductive and deductive approaches.

Ethics and dissemination: The study was approved by the WHO/HRP Research Project Review Panel and WHO Ethical Review Committee (A65932) as well as the Health Research Ethics Committee, Faculty of Medicine, Universitas Mataram in Indonesia (004/UN18/F7/ETIK/2023).

Findings will be disseminated through research publications and communicated to the Lombok district health offices. The analyses from this study will also inform the design of a subsequent impact evaluation.

Strengths and Limitations:

- This study addresses a research gap identified by the World Health Organization and expands on the geographic scope of available evidence on SMBP beyond high-income settings.
- The study also uses a novel approach of a smartphone-based BP measurement to examine the intersection of digital health, maternal health, self-care, and health systems.
- To provide a holistic perspective on the barriers and operational requirements for SMBP, the study will include a diverse set of stakeholders of pregnant women, their partners, and health workforce across different levels of the health system.
- A limitation of this study is that pregnant women will not be actively self-monitoring their BP to detect clinical changes but rather undergo the process to understand the perceptions of SMBP and implementation considerations.
- This study will inform subsequent impact evaluations and compliments existing initiatives within the research site on the use of digital tools towards identification and management of HDP.

For peer review only - http://bmjopen.bmj.com/site/about/guidelines.xhtml

Introduction

Hypertensive disorders of pregnancy (HDPs) are one of the most common pregnancy complications and a leading cause of maternal mortality and morbidity.(1-5) Approximately 14 percent of all maternal deaths globally are attributed to HDPs, with the majority in low and middle income contexts.(2, 3) HDPs include chronic hypertension, gestational hypertension, preeclampsia, and chronic hypertension with superimposed preeclampsia.(1, 5) Chronic hypertension consists of elevated blood pressure (BP) that is identified prior to the pregnancy or detected within the first 20 weeks.(1) Gestational hypertension is considered to be induced during a pregnancy with the onset of elevated BP after 20 weeks. (1) Pre-eclampsia is characterized by elevated blood pressure in the presence of either proteinuria or other new onset maternal organ dysfunction, neurological conditions, or fetal growth restriction.(6) It is a precursor to eclampsia, a life-threatening condition which accounts for the majority of deaths attributed to HDPs.(2, 4)

The World Health Organization (WHO) recommends routine BP measurement during pregnancy and at every point of contact in the provision of antenatal care (ANC) as part of efforts to improve maternal health.(7, 8) In certain contexts, pregnant women identified with HDP may require additional follow-up and monitoring in order to mitigate potential adverse outcomes.(9) Furthermore, as the underlying cause of pre-eclampsia is unknown and onset may be triggered in between clinical consultations, close monitoring of BP beyond the health facility is critical. (5, 10, 11)

Self-monitoring of blood pressure

Self-monitoring of blood pressure (SMBP), also known as home-based BP monitoring, is well researched in the general population (12-17) with a WHO recommendation in 2013 supporting its appropriate and affordable use.(18) Cited advantages include improved BP control and reduced anxiety associated with 'white coat' hypertension. (10-12, 15, 17) However, studies also highlight challenges associated with the variety of devices used within home settings, reliability of readings and calibration requirements, as well as ensuring guidance to individuals on interpreting results, dealing with variations of readings, and appropriate actions to be taken. (19, 20)

SMBP during pregnancy is an emerging approach recommended by WHO in 2021 "as an additional option to clinic blood pressure monitoring by health workers during antenatal contacts only, for individuals with hypertensive disorders of pregnancy."(21) Although there is a substantial literature base on SMBP in non-pregnant populations (12-16, 19, 20, 22-29), the evidence specific to pregnancy is still nascent (11, 30-33) and limited for low- and middle-income countries (LMIC) where there remains a high burden of HDPs.(34) To date, studies that explore SMBP among pregnant women span Australia, Canada, Japan, United Kingdom (UK), New Zealand, and United States (US) (35, 36), with one study in South Africa focused on knowledge and attitudes of SMBP among preeclamptic patients.(34) A Cochrane systematic review on the different approaches to SMBP in pregnancy indicated uncertainties in the benefits of SMBP of hypertensive pregnancies due to limited number of studies.(37) Other systematic reviews on SMBP during antenatal and postpartum periods demonstrated its feasibility but no difference in clinical outcomes (38), while another metanalyses reported reductions in antenatal care visits, hospital admissions, and diagnosis of preeclampsia (39). All systematic reviews highlighted the lack of studies from LMICs and the need for more evidence, particularly across diverse settings. (37-39)

Among the available studies, the Blood Pressure Self-Monitoring in Pregnancy (BuMP) research initiative conducted in the UK, offers one of the most comprehensive methods for evaluating feasibility and effectiveness. (30, 31, 40, 41) For the feasibility assessment, women between 12-16 weeks gestational age and identified to be at risk for pre-eclampsia were recruited to self-monitor their BP using an automated BP monitor (Microlife WatchBP Home validated for use in pregnancy and pre-eclampsia).

For peer review only - http://bmjopen.bmj.com/site/about/guidelines.xhtml

Study participants had the option to also text their readings and receive automated responses and text prompts. (31) Women reported that the approach was reassuring and made them more informed about HDPs; however, study authors noted the limited generalizability of the findings across different socioeconomic backgrounds.(31) This assessment was followed by a prospective cohort study of approximately 200 women, demonstrating that women at risk for pre-eclampsia were able to obtain more frequent BP readings and facilitate the detection of gestational hypertension in-between clinic visits.(30) The study also explored healthcare providers' perspectives and noted their concerns in reconciling the autonomy and reassurance that women may gain in exchange with the caution and uncertainty that self-monitoring requires.(41) More recently, the BuMP study conducted a randomized clinical trial (RCT) among 2441 pregnant individuals at increased risk for preeclampsia, in which there was no statistically significant differences in early detection of preeclampsia between SMBP and standard care groups (42). Similarly, there was also no statistically significant differences among pregnant individuals with chronic or gestational hypertension.(43)

Another RCT, also conducted in the UK, enrolled pregnant women with chronic hypertension or gestational hypertension for SMBP using a calibrated BP measurement device (Microlife WatchBP Home); women with pre-eclampsia were excluded from this study. (44) Study participants reported their second of two BP readings via text, an app designed for the study, or noted it in on paper to be reviewed at their ANC contact.(44) In response to their text message or the mobile application, participants received immediate automated responses. Although there was no difference in the mean systolic BP measurements or medication adherence, participants demonstrated willingness and acceptability for self-monitoring.(44)

Potential for digital technology to facilitate SMBP

Advances in digital technology in LMICs, primarily through penetration of mobile devices(45), provide an opportunity to expand SMBP and other health monitoring services. While the catalyst for SBPM in high income settings was spurred by the rise of automated cuffs(12), this has not gained traction in settings in which there is already a limited availability of automated equipment for facility based use.(46) Furthermore, BP measurement devices require additional validation specific to pregnant populations.(47, 48)

As an emerging alternative to measuring BP, OptiBP™ is a software application that utilizes smartphone cameras to capture optical pulse-waves of the fingertip and estimate BP readings (Figure 1). The OptiBP™ application runs on Android OS 8.1 and leverages the smartphone camera to record photoplethysmographic (PPG) optical pulse waves derived from blood volume changes at the fingertips and estimate BP values using an algorithm.(49, 50). The OptiBP™ application has undergone validation in Switzerland, in which it demonstrated concordance against reference BP measurements; (50, 51) A complementary validation study of OptiBP was conducted in accordance with the international regulatory standards in Tanzania, South Africa, and Bangladesh. This study also included a targeted assessment on the accuracy of BP measurement among pregnant populations to account for the maternal cardiovascular changes on the detection of optical pulse waves.(52, 53) Findings from this multisite study demonstrated accuracy against International Organization for Standardization (ISO) benchmarks, including in pregnant populations, except in Bangladesh for systolic blood pressure ISO criterion 2.(53, 54) A similar validation study was also conducted in Indonesia among pregnant populations in West Lombok and East Lombok in West Nusa Tenggara Province.(55)

Figure 1 Optical pulse wave technology underlying OptiBP smartphone application(50)

For peer review only - http://bmjopen.bmj.com/site/about/guidelines.xhtml

Aims and Objectives

The newfound capability of using a smartphone application for BP measurement presents an opportunity for broadening SMBP to LMIC settings, where coverage of health services is more limited and mobile technologies have gained widespread use for maternal health.(52, 56-64) Using these advances in BP monitoring, this research addresses evidence gaps identified in the WHO guideline on self-care interventions regarding the feasibility, benefits, and harms of SMBP among individuals with HDP.(21) This study aims to understand the perceptions, barriers, and operational considerations for SMBP via a smartphone application (OptiBP) in primary health care settings of Lombok, Indonesia. The first objective of this study is to explore pregnant women's, their partners, and health workers' attitudes towards SMBP in general, and specifically regarding the use of a smartphone application for SMBP. In addition, the study will include health system managers and examine the implementation requirements through user-centered design approaches to adapt for health literacy and workflow integration requirements.

Methods and analysis:

Study design and setting

This study will use a combination of qualitative methods, including focus group discussions (FGD), in-depth interview (IDI), and workshop observations to triangulate findings on perceptions and operational considerations for SMBP by pregnant women. The study will include pregnant women, their partners, and the health workers involved in the provision and management of ANC services to obtain a holistic understanding of the dynamics across health system actors. To derive concrete operational considerations, selected pregnant women will also be provided with a research version of the OptiBP™ application to use in their home setting and subsequently provide feedback on their experiences. The study will be conducted within the catchment area of six primary health care facilities in West Lombok district Lombok, Indonesia. Data collection is started in mid-February 2023 and expected to be completed by May 2023.

Participant selection

The main population for this study is pregnant women with current or past hypertensive disorders of pregnancy. Eligible participants will be drawn from those pregnant women coming to receive ANC at the selected study facilities and registered in the ANC digital module, which is a digital health record tracking and decision support system that was implemented independently of this study.(65) Following approval of requests from the heads of health facilities to review the digital health records for sampling purposes, the study team will select pregnant women for screening and invitation to participate in the study. A subset of partners of the pregnant women included in the study will also be selected following the consent of the pregnant woman to contact her partner.

A diverse range of health workforce cadres, including community health workers, community and facility-based midwives, obstetrician/gynecologists, and health system managers will also be included to compliment the findings from pregnant women. Table 1 provides an overview of the participants and sampling frame.

Table 1. Sampling frame

For peer review only - http://bmjopen.bmj.com/site/about/guidelines.xhtml

Activity/Method	Pregnant women with HDP		Partners of pregnant women	Health workers/ manager				
	<30 years	>=30 years		CHWs	Nurse-midwives (community)	Nurse-midwives (facility)	Doctors	Health system managers
FGD on perception of SMBP	2-3 FGDs of 5-7	2-3 FGDs of 5-7	2 FGDs of 5-7	4 FGDs of 5-7	2-3 FGDs of 5-7	2-3 FGDs of 5-7	N/A	N/A
IDI on perception of SMBP	4-5	4-5	N/A	4-5	4-5	4-5	3-5	3-5
Intervention adaptation workshops	5-10	5-10	N/A	N/A	N/A	N/A	N/A	N/A
FGD: Focus Group Discussion; IDI: In-depth Interview; HDP: Hypertensive diseases in pregnancy; N/A: Not applicable								

Sampling and recruitment

The study will use purposive sampling of pregnant women with a current diagnosis or history of HDP (as derived from their medical record in the ANC digital module) and across two age groups to account for potentially differing levels of digital literacy. We will identify women with different educational levels, gestational age, and parity through the sampling and collect variables on these factors.

Pregnant women will be approached during their routine ANC contacts once they have completed their consultation. A research assistant will brief women on the study and gauge their interest to participate. The contact information of consenting and eligible pregnant women will be taken, and the research assistant will inform them that they will be contacted if they are selected for the study. Eligibility for pregnant participants will be based on the following criteria: confirmed pregnancy (either by health worker or self-administered and documented rapid test), current or history of HDP (derived from the digital record system), greater than 18 years of age or emancipated minor, living within catchment area of selected health facilities, able to read one sentence in Bahasa, and having provided written informed consent. Pregnant women will be excluded if they have severe visual impairment that prevents use of the smartphone application (e.g., blindness) or absence of index or middle finger on either left or right hand. Partners of pregnant women will be identified through the recruited women after first obtaining consent from the pregnant women to contact their partners. Pregnant women will not be excluded based on ownership of a phone, and the study will provide a smartphone to the participating women who are assigned to use the application in their home setting.

Healthcare managers will be selected based on their role and oversight in the catchment area. They will be invited to participate in the study and asked for their written informed consent during their work hours. The different cadres of health workers will be identified through District Health Office (DHO) and facility networks under its hierarchical systems, which are Public Health Centers (Puskesmas) and Maternity Clinics (Polindes).

Health workers (nurse-midwives and community health workers) will be randomly selected to participate in the study based on a list provided by the DHO. The study team will first briefly inform by the supervisors and invite the selected health workers to attend an information session where the research

For peer review only - http://bmjopen.bmj.com/site/about/guidelines.xhtml

staff will elaborate further on the study and ask for their consent to take part in the study. The interviews will take place during work hours after permission has been obtained from the DHO.

The study will invite women to either be part of the FGDs or workshops and randomly allocate to the two groups. Participants for the IDIs will be sampled from the FGDs to examine their feedback in greater detail. The sample size for pregnant women and health workers will be based on requirements for reaching saturation, as well as using an emergent sampling design, in which participants will be recruited as the study progresses.(66)

Methods of approach

Interviews

FGDs and IDIs will be used to explore potential barriers and motivators for SMBP, as well as how this may influence changes in pregnant women's interactions with health workers. As partners and family members of pregnant women can influence access and use of ANC services, their perceptions of SMBP will also be used to obtain a comprehensive understanding of the considerations for SMBP and any related care.(67)

Observation workshops

In addition to the FGDs and IDIs, observation workshops will be held to review the smartphone application interface and determine adaptation requirements for enhancing usability in accordance with health literacy levels and ensuring integration with health system and data workflows. The workshop will be conducted in two parts. First, we will introduce a research version of the smartphone application to selected women and observe initial reactions and feedback. This version of the application would be linked to a de-identified study account and include a disclaimer stating the measurements are not to be used for clinical decision-making and that participants should contact health facility and research team if they face any health issues. The application will not be used by the research team or health workers for any clinical decision making, and all BP measurements for service provision will be done through the standard care at facilities using standard sphygmomanometers. Women will be provided with a smartphone with the application and instructions to explore using it at home twice a day for one week before returning to the subsequent workshop, one week later. This second workshop will be used to gather feedback on their experiences of use and refine guidance/training support needed for achieving fidelity and requirements for integrating within health system workflows.

Both workshops will include structured and unstructured observations of pregnant women engaging with the smartphone application interface and will be used to further inform the decision support prompts (e.g., what to do in cases of high BP reading) that are contextually appropriate and resonate with the levels of health literacy. In the structured observations, pregnant women will be asked to interact with the smartphone application prototype across various tasks and scenarios, such as turning on the smartphone application, placing their finger on the camera of the smartphone for taking their BP measurement, interpreting the result/output from the BP measurement, and undertaking follow-up actions, when necessary.

Data collection

For the interviews, a semi-structured topic guide will be provided to trained interviewers conducting the FGDs and IDIs. The topic guide for pregnant individuals will cover areas of pregnancy experience, mobile phone use in pregnancy, knowledge of and experience with taking BP, self-efficacy in taking BP, trust, and support for taking BP. Interview guides for the health worker will include topics surrounding experience in providing ANC, perceptions of self-management of pregnancy generally, perceptions of SMBP by pregnant women, and data flow and health system linkages. All interview guides will include

For peer review only - http://bmjopen.bmj.com/site/about/guidelines.xhtml

probes and questions for initiating the interviews and allow for open-ended responses and capturing any reflections that may emerge. Interviews will be recorded, transcribed, and translated into English for analysis.

For the observation workshops, a research assistant will document both unprompted reactions, as well as responses to specific tasks with the smartphone application. As a supplement to the observation notes, study will also embed a screen recorder while pregnant women use the application during the workshop to capture the process flows and interactions with the application, including length of time and frequency of use of different buttons. The study team will also take videos and photos during the observation workshop to record feedback related to interactions with the application.

Analysis

Recordings will be double transcribed and translated for quality assurance. Data obtained from the interviews and observations will first undergo an initial rapid analysis to flag key themes and considerations, including findings from the existing literature base. This will be followed by detailed analysis and coding using a thematic analysis employing a combination of both inductive and deductive approaches. (66, 68) Each finding will be compared and crosschecked to determine whether it aligns with existing categories or if a new theme should be developed.(66) The analysis will be conducted by two individuals to ensure interrater reliability and then reviewed collectively with the broader research team. Screen recordings of interactions with the application will also be reviewed to identify patterns of use and ATLAS.ti (69) will be used to manage the coding and analysis of themes.

Patient and public involvement

The views of patients or the public will be reflected in the analysis of the manuscript and through the co-design workshops of the study.

Ethics and dissemination:

The study was approved by the WHO/HRP Research Review Panel and WHO Ethical Review Committee (Reference number A65932) as well as the Health Research Ethics Committee, Faculty of Medicine, Universitas Mataram in Indonesia (004/UN18/F7/ETIK/2023).

All eligible participants will receive an information sheet and required to provide informed written consent before participation. Confidential data will be stored at country level and de-identified transcriptions will be shared with WHO to support the analyses. Personal identifiers will be removed during the transcription of audio recordings and labelled with a de-identified participant identification number. Identifiable data will be kept at the research site in a secure location for a minimum of five years before securely disposing of primary research data, as per the guidance from the local ethical review committee.

Participants using the smartphone application at home will be provided with detailed guidance, as well as airtime/SIM cards to contact the research team as needed. The smartphone application will not be used by the research team or health workers for any clinical decision making, and all BP measurements for service provision will be done through the standard care at facilities using standard sphygmomanometers.

Photos and video recordings will only be taken during trainings and specific group events, and only after written informed consent. The informed consent will explicitly mention the use of audio-recording and screen recording during the workshop. Unless otherwise consented, use of these will be obscured by blurring or other effects to render persons non-identifiable, and the purpose for use will be restricted to training and implementing processes to make the work or intervention more effective. The company that

For peer review only - http://bmjopen.bmj.com/site/about/guidelines.xhtml

developed the smartphone application will not have access to any data or transcripts but will have access to the feedback shared during their participation in the workshop.

Findings will be disseminated through research publications and communicated to the DHO. The analyses from this study will also inform the design of a subsequent impact evaluation. In addition, the feedback from workshops may contribute to potential refinements to the smartphone application.

Authors' contributions: TT, YS, MB, AS, and OT developed the protocol from which excerpts were used to draft this manuscript. AG, MG, MPP, and SOR reviewed the protocol and provided comments that fed into the manuscript. TT prepared the first draft of the manuscript. All authors reviewed both the protocol and this manuscript. AT and AS are shared senior authors.

Acknowledgements: We thank the heads of health centers from the study sites (Penimbung, Ranjok, Jembatan Kembar, Narmada Gerung, and Meninting) support to conduct the study. We are also grateful to Dr Garrett Mehl (WHO) for support in conceptualization of the study.

Funding: This work was funded by the Bill and Melinda Gates Foundation (grant number OPP1201339) and the UNDP-UNFPA-UNICEF-WHO-World Bank Special Programme of Research, Development and Research Training in Human Reproduction (HRP), a cosponsored programme executed by the World Health Organization (WHO). This research received no specific grant from any funding agency in the public, commercial or not-for-profit sectors.

Competing interests: None declared from authors. Biospectal, the company that developed the OptiBP application, will provide a research version of the application for use in the study but did not play a role in the study design.

Disclaimer: The authors alone are responsible for the views expressed in this article and they do not necessarily represent the views, decisions, or policies of the institutions with which they are affiliated.

References:

1. Mammaro A, Carrara S, Cavaliere A, Ermito S, Dinatale A, Pappalardo EM, et al. Hypertensive disorders of pregnancy. J Prenat Med. 2009;3(1):1-5.
2. Duley L. The global impact of pre-eclampsia and eclampsia. Semin Perinatol. 2009;33(3):130-7.
3. Say L, Chou D, Gemmill A, Tunçalp Ö, Moller A-B, Daniels J, et al. Global causes of maternal death: a WHO systematic analysis. The Lancet Global Health. 2014;2(6):e323-e33.
4. Abalos E, Cuesta C, Grosso AL, Chou D, Say L. Global and regional estimates of preeclampsia and eclampsia: a systematic review. Eur J Obstet Gynecol Reprod Biol. 2013;170(1):1-7.
5. World health Organization. WHO Recommendations for Prevention and Treatment of Pre-Eclampsia and Eclampsia. WHO Guidelines Approved by the Guidelines Review Committee. Geneva2011.
6. JA24 Pre-eclampsia. In: ICD-11 for mortality and morbidity statistics. Geneva: World Health Organization. (https://icd.who.int/ browse11/l-m/en#/http://id.who.int/icd/entity/229121159, accessed 2 July 2023).
7. Tsakane MAG Hlongwane BB, Maria C Barreix, Robert Pattinson, Metin Gülmezoglu, Valerie Vannevel , Özge Tunçalp. Implementing antenatal care recommendations, South Africa. Bulletin of the World Health Organization. 2021;99::220-7.
8. World Health Organization. WHO Recommendations on antenatal care for a positive pregnancy experience. Geneva: WHO; 2016.

For peer review only - http://bmjopen.bmj.com/site/about/guidelines.xhtml

9. Excellence NIfHaC. Hypertension in pregnancy: diagnosis and management -- NICE guideline [NG133] United Kingdom2019 [Available from: https://www.nice.org.uk/guidance/ng133/chapter/Recommendations.
10. Mustafa R, Ahmed S, Gupta A, Venuto RC. A comprehensive review of hypertension in pregnancy. J Pregnancy. 2012;2012:105918.
11. Hodgkinson JA, Tucker KL, Crawford C, Greenfield SM, Heneghan C, Hinton L, et al. Is self monitoring of blood pressure in pregnancy safe and effective? BMJ. 2014;349:g6616.
12. Parati G, Stergiou GS, Asmar R, Bilo G, de Leeuw P, Imai Y, et al. European Society of Hypertension practice guidelines for home blood pressure monitoring. J Hum Hypertens. 2010;24(12):779-85.
13. Imai Y, Obara T, Asamaya K, Ohkubo T. The reason why home blood pressure measurements are preferred over clinic or ambulatory blood pressure in Japan. Hypertens Res. 2013;36(8):661-72.
14. Roerecke M, Kaczorowski J, Myers MG. Comparing Automated Office Blood Pressure Readings With Other Methods of Blood Pressure Measurement for Identifying Patients With Possible Hypertension: A Systematic Review and Meta-analysis. (2168-6114 (Electronic)).
15. Bo Y, Kwok KO, Chu KK, Leung EY, Yu CP, Wong SY, et al. Comparison Between Automated Office Blood Pressure Measurements and Manual Office Blood Pressure Measurements-Implications in Individual Patients: a Systematic Review and Meta-analysis. (1534-3111 (Electronic)).
16. Niiranen TJ, Jula Am Fau - Kantola IM, Kantola Im Fau - Reunanen A, Reunanen A. Comparison of agreement between clinic and home-measured blood pressure in the Finnish population: the Finn-HOME Study. (0263-6352 (Print)).
17. McManus RJ, Little P, Stuart B, Morton K, Raftery J, Kelly J, et al. Home and Online Management and Evaluation of Blood Pressure (HOME BP) using a digital intervention in poorly controlled hypertension: randomised controlled trial. Bmj. 2021;372:m4858.
18. World Health Organization. Package of Essential Noncommunicable (PEN) disease interventions for primary health care in low-resource settings. Geneva, Switzerland2013.
19. Fletcher BR, Hinton L, Hartmann-Boyce J, Roberts NW, Bobrovitz N, McManus RJ. Self-monitoring blood pressure in hypertension, patient and provider perspectives: A systematic review and thematic synthesis. Patient education and counseling. 2016;99(2):210-9.
20. McManus RJ, Ryan A, Greenfield S, Pattison HM, Clifford S, Marriott J, et al. Self measurement of blood pressure: a community survey. Journal of Human Hypertension. 2007;21(9):741-3.
21. World Health Organization. WHO Consolidated Guideline on Self-Care Interventions for Health v.2 Sexual and Reproductive Health and Rights Geneva: World Health Organization; 2022 [Available from: https://www.who.int/publications/i/item/9789240052192.
22. Monahan M, Jowett S, Lovibond K, Gill P, Godwin M, Greenfield S, et al. Predicting Out-of-Office Blood Pressure in the Clinic for the Diagnosis of Hypertension in Primary Care: An Economic Evaluation. Hypertension. 2018;71(2):250-61.
23. Hodgkinson J, Mant J, Martin U, Guo B, Hobbs FD, Deeks JJ, et al. Relative effectiveness of clinic and home blood pressure monitoring compared with ambulatory blood pressure monitoring in diagnosis of hypertension: systematic review. BMJ. 2011;342:d3621.
24. Beyhaghi H, Viera AJ. Comparative Cost-Effectiveness of Clinic, Home, or Ambulatory Blood Pressure Measurement for Hypertension Diagnosis in US Adults. Hypertension. 2019;73(1):121-31.
25. Tucker K, Sheppard JP, Stevens R, Bosworth HB, Bove A, Bray EP, et al. [OP.6B.04] INDIVIDUAL PATIENT DATA META-ANALYSIS OF SELF-MONITORING OF BLOOD PRESSURE (BP-SMART). Journal of Hypertension. 2016;34.
26. Bray EP, Holder R, Mant J, McManus RJ. Does self-monitoring reduce blood pressure? Meta-analysis with meta-regression of randomized controlled trials. Database of Abstracts of Reviews of Effects (DARE). 2011.

For peer review only - http://bmjopen.bmj.com/site/about/guidelines.xhtml

27. Uhlig K, Patel K, Ip S, Kitsios GD, Balk EM. Self-Measured Blood Pressure Monitoring in the Management of Hypertension: A Systematic Review and Meta-analysis. Annals of internal medicine. 2013;159(3):185-94.
28. Ward AM, Takahashi O, Stevens R, Heneghan C. Home measurement of blood pressure and cardiovascular disease: systematic review and meta-analysis of prospective studies. J Hypertens. 2012;30(3):449-56.
29. Niiranen TJ, Hänninen Mr Fau - Johansson J, Johansson J Fau - Reunanen A, Reunanen A Fau - Jula AM, Jula AM. Home-measured blood pressure is a stronger predictor of cardiovascular risk than office blood pressure: the Finn-Home study. (1524-4563 (Electronic)).
30. Tucker KL, Taylor KS, Crawford C, Hodgkinson JA, Bankhead C, Carver T, et al. Blood pressure self-monitoring in pregnancy: examining feasibility in a prospective cohort study. BMC Pregnancy Childbirth. 2017;17(1):442.
31. Hinton L, Tucker KL, Greenfield SM, Hodgkinson JA, Mackillop L, McCourt C, et al. Blood pressure self-monitoring in pregnancy (BuMP) feasibility study; a qualitative analysis of women's experiences of self-monitoring. BMC Pregnancy Childbirth. 2017;17(1):427.
32. Tucker KL, Bowen L, Crawford C, Mallon P, Hinton L, Lee MM, et al. The feasibility and acceptability of self-testing for proteinuria during pregnancy: A mixed methods approach. Pregnancy Hypertens. 2018;12:161-8.
33. Wilson H, Tucker KL, Chisholm A, Hodgkinson J, Lavallee L, Mackillop L, et al. Self-monitoring of blood pressure in pregnancy: A mixed methods evaluation of a national roll-out in the context of a pandemic. Pregnancy Hypertension. 2022;30:7-12.
34. Munyungula J, Shakwane S. Self-monitoring of blood pressure for preeclampsia patients: Knowledge and attitudes. Curationis. 2021;44(1):e1-e8.
35. Tucker KL, Bankhead C, Hodgkinson J, Roberts N, Stevens R, Heneghan C, et al. How Do Home and Clinic Blood Pressure Readings Compare in Pregnancy? Hypertension. 2018;72(3):686-94.
36. Aquino M, Munce S, Griffith J, Pakosh M, Munnery M, Seto E. Exploring the Use of Telemonitoring for Patients at High Risk for Hypertensive Disorders of Pregnancy in the Antepartum and Postpartum Periods: Scoping Review. JMIR Mhealth Uhealth. 2020;8(4):e15095.
37. Ashworth DC, Maule SP, Stewart F, Nathan HL, Shennan AH, Chappell LC. Setting and techniques for monitoring blood pressure during pregnancy. Cochrane Database Syst Rev. 2020;8(8):Cd012739.
38. Yeh PT, Rhee DK, Kennedy CE, Zera CA, Lucido B, Tuncalp O, et al. Self-monitoring of blood pressure among women with hypertensive disorders of pregnancy: a systematic review. BMC Pregnancy Childbirth. 2022;22(1):454.
39. Kalafat E, Benlioglu C, Thilaganathan B, Khalil A. Home blood pressure monitoring in the antenatal and postpartum period: A systematic review meta-analysis. Pregnancy Hypertens. 2020;19:44-51.
40. Dougall G, Franssen M, Tucker KL, Yu LM, Hinton L, Rivero-Arias O, et al. Blood pressure monitoring in high-risk pregnancy to improve the detection and monitoring of hypertension (the BUMP 1 and 2 trials): protocol for two linked randomised controlled trials. BMJ Open. 2020;10(1):e034593.
41. Hinton L, Hodgkinson J, Tucker KL, Rozmovits L, Chappell L, Greenfield S, et al. Exploring the potential for introducing home monitoring of blood pressure during pregnancy into maternity care: current views and experiences of staff-a qualitative study. BMJ Open. 2020;10(12):e037874.
42. Tucker KL, Mort S, Yu LM, Campbell H, Rivero-Arias O, Wilson HM, et al. Effect of Self-monitoring of Blood Pressure on Diagnosis of Hypertension During Higher-Risk Pregnancy: The BUMP 1 Randomized Clinical Trial. Jama. 2022;327(17):1656-65.
43. Chappell LC, Tucker KL, Galal U, Yu LM, Campbell H, Rivero-Arias O, et al. Effect of Self-monitoring of Blood Pressure on Blood Pressure Control in Pregnant Individuals With Chronic or Gestational Hypertension: The BUMP 2 Randomized Clinical Trial. Jama. 2022;327(17):1666-78.

For peer review only - http://bmjopen.bmj.com/site/about/guidelines.xhtml

44. Pealing LM, Tucker KL, Mackillop LH, Crawford C, Wilson H, Nickless A, et al. A randomised controlled trial of blood pressure self-monitoring in the management of hypertensive pregnancy. OPTIMUM-BP: A feasibility trial. Pregnancy Hypertens. 2019;18:141-9.
45. International Telecommunications Union. ICT Facts and Figures. Geneva: 2015 (Avaliable from https://www.itu.int/en/ITU-D/Statistics/Documents/facts/ICTFactsFigures2015.pdf, accessed 23 March 2018)
46. Betran AP, Bergel E, Griffin S, Melo A, Nguyen MH, Carbonell A, et al. Provision of medical supply kits to improve quality of antenatal care in Mozambique: a stepped-wedge cluster randomised trial. Lancet Glob Health. 2018;6(1):e57-e65.
47. Ashworth DC, Maule SP, Stewart F, Nathan HL, Shennan AH, Chappell LC. Setting and techniques for monitoring blood pressure during pregnancy. Cochrane Database Syst Rev. 2020;8:CD012739.
48. O'Brien E, Atkins N, Stergiou G, Karpettas N, Parati G, Asmar R, et al. European Society of Hypertension International Protocol revision 2010 for the validation of blood pressure measuring devices in adults. Blood Pressure Monitoring. 2010;15(1):23-38.
49. Ghamri Y, Proença M, Hofmann G, Renevey P, Bonnier G, Braun F, et al. Automated Pulse Oximeter Waveform Analysis to Track Changes in Blood Pressure During Anesthesia Induction: A Proof-of-Concept Study. Anesthesia & Analgesia. 2020;130(5).
50. Schoettker P, Degott J, Hofmann G, Proença M, Bonnier G, Lemkaddem A, et al. Blood pressure measurements with the OptiBP smartphone app validated against reference auscultatory measurements. Scientific Reports. 2020;10(1):17827.
51. Degott J, Ghajarzadeh-Wurzner A, Hofmann G, Proença M, Bonnier G, Lemkaddem A, et al. Smartphone based blood pressure measurement: accuracy of the OptiBP mobile application according to the AAMI/ESH/ISO universal validation protocol. Blood Pressure Monitoring. 2021.
52. Su F, Li Z, Sun X, Han N, Wang L, Luo X. The pulse wave analysis of normal pregnancy: investigating the gestational effects on photoplethysmographic signals. Biomed Mater Eng. 2014;24(1):209-19.
53. Association for the Advancement of Medical Instrumentation, Association for the Advancement of Medical Instrumentation, European Society of Hypertension, International Organization for Standardization (AAMI/ESH/ISO). Non-invasive sphygmomanometers — Part 2: Clinical investigation of automated measurement type. Arlington, VA, USA; 2013.
54. Festo C, Vannevel V, Ali H, Tamrat T, Mollel GJ, Hlongwane T, et al. Accuracy of a smartphone application for blood pressure estimation in Bangladesh, South Africa, and Tanzania. npj Digital Medicine. 2023;6(1):69.
55. Setiyawati YD, Jannah M, Gayatri M, Meylentia E, Shankar AH (In Press) The accuracy of cuffless optical blood pressure assessment in pregnancy and its potential to improve clinical outcomes.
56. Saronga NJ, Burrows T, Collins CE, Ashman AM, Rollo ME. mHealth interventions targeting pregnancy intakes in low and lower-middle income countries: Systematic review. Matern Child Nutr. 2019;15(2):e12777.
57. Ilozumba O, Abejirinde IO, Dieleman M, Bardaji A, Broerse JEW, Van Belle S. Targeting strategies of mHealth interventions for maternal health in low and middle-income countries: a systematic review protocol. BMJ Open. 2018;8(2):e019345.
58. Dol J, Campbell-Yeo M, Tomblin Murphy G, Aston M, McMillan D, Richardson B. Impact of mobile health interventions during the perinatal period for mothers in low- and middle-income countries: a systematic review protocol. JBI Database System Rev Implement Rep. 2019;17(2):137-46.
59. Chen H, Chai Y, Dong L, Niu W, Zhang P. Effectiveness and Appropriateness of mHealth Interventions for Maternal and Child Health: Systematic Review. JMIR Mhealth Uhealth. 2018;6(1):e7.
60. Watterson JL, Walsh J, Madeka I. Using mHealth to Improve Usage of Antenatal Care, Postnatal Care, and Immunization: A Systematic Review of the Literature. Biomed Res Int. 2015;2015:153402.

For peer review only - http://bmjopen.bmj.com/site/about/guidelines.xhtml

61. Sondaal SF, Browne JL, Amoakoh-Coleman M, Borgstein A, Miltenburg AS, Verwijs M, et al. Assessing the Effect of mHealth Interventions in Improving Maternal and Neonatal Care in Low- and Middle-Income Countries: A Systematic Review. PLoS One. 2016;11(5):e0154664.

62. Nurmatov UB, Lee SH, Nwaru BI, Mukherjee M, Grant L, Pagliari C. The effectiveness of mHealth interventions for maternal, newborn and child health in low- and middle-income countries: Protocol for a systematic review and meta-analysis. J Glob Health. 2014;4(1):010407.

63. Lee SH, Nurmatov UB, Nwaru BI, Mukherjee M, Grant L, Pagliari C. Effectiveness of mHealth interventions for maternal, newborn and child health in low- and middle-income countries: Systematic review and meta-analysis. J Glob Health. 2016;6(1):010401.

64. World Health Organization. WHO guideline: Recommendations on digital interventions for health system strengthening Geneva: World Health Organization; 2019 [124]. Available from: https://www.who.int/publications/i/item/who-digital-implementation-investment-guide.

65. Haddad SM, Souza RT, Cecatti JG, Barreix M, Tamrat T, Footitt C, et al. Building a Digital Tool for the Adoption of the World Health Organization's Antenatal Care Recommendations: Methodological Intersection of Evidence, Clinical Logic, and Digital Technology. J Med Internet Res. 2020;22(10):e16355.

66. Patton MQ. Qualitative evaluation checklist: Evaluation Checklist Project; 2013 [Available from: https://wmich.edu/sites/default/files/attachments/u350/2014/qualitativeevalchecklist.pdf.

67. Suandi D, Williams P, Bhattacharya S. Does involving male partners in antenatal care improve healthcare utilisation? Systematic review and meta-analysis of the published literature from low- and middle-income countries. Int Health. 2020;12(5):484-98.

68. Pope C, Ziebland S, Mays N. Qualitative research in health care. Analysing qualitative data. BMJ. 2000;320(7227):114-6.

69. ATLAS.ti Scientific Software Development GmbH. ATLAS.ti 23.2.1. ATLAS.ti Scientific Software Development GmbH. 2023.

For peer review only - http://bmjopen.bmj.com/site/about/guidelines.xhtml

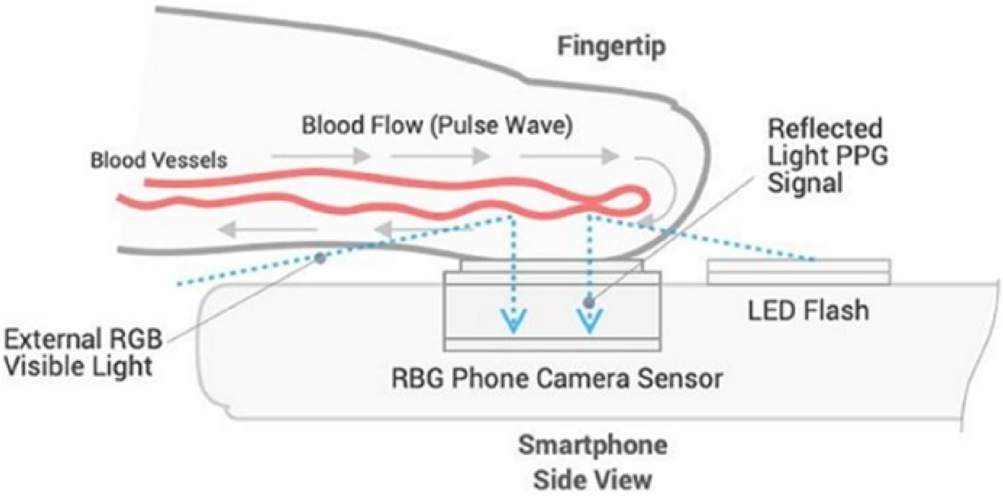

Figure 1 Optical pulse wave technology underlying OptiBP smartphone application(50)

79x39mm (220 x 220 DPI)
